# Fundamentals, Algorithms, and Technologies of Occupancy Detection for Smart Buildings Using IoT Sensors

**DOI:** 10.3390/s24072123

**Published:** 2024-03-26

**Authors:** Pratiksha Chaudhari, Yang Xiao, Mark Ming-Cheng Cheng, Tieshan Li

**Affiliations:** 1 Department of Computer Science, University of Alabama, Tuscaloosa, AL 35487, USA; pgchaudhari@crimson.ua.edu; 2 Department of Electrical and Computer Engineering, University of Alabama, Tuscaloosa, AL 35487, USA; mmcheng@ua.edu; 3 School of Automation Engineering, University of Electronic Science and Technology of China, Chengdu 611731, China; tieshanli@126.com

**Keywords:** occupancy detection algorithms, smart buildings, IoT sensors, devices, machine learning

## Abstract

Smart buildings use advanced technologies to automate building functions. One important function is occupancy detection using Internet of Things (IoT) sensors for smart buildings. Occupancy information is useful information to reduce energy consumption by automating building functions such as lighting, heating, ventilation, and air conditioning systems. The information is useful to improve indoor air quality by ensuring that ventilation systems are used only when and where they are needed. Additionally, it is useful to enhance building security by detecting unusual or unexpected occupancy levels and triggering appropriate responses, such as alarms or alerts. Occupancy information is useful for many other applications, such as emergency response, plug load energy management, point-of-interest identification, etc. However, the accuracy of occupancy detection is limited by factors such as real-time occupancy data, sensor placement, privacy concerns, and the presence of pets or objects that can interfere with sensor reading. With the rapid development of IoT sensor technologies and the increasing need for smart building solutions, there is a growing interest in occupancy detection techniques. There is a need to provide a comprehensive survey of these technologies. Although there are some exciting survey papers, they all have limited scopes with different focuses. Therefore, this paper provides a comprehensive overview of the current state-of-the-art occupancy detection methods (including both traditional algorithms and machine learning algorithms) and devices with their advantages and limitations. It surveys and compares fundamental technologies (such as sensors, algorithms, etc.) for smart buildings. Furthermore, the survey provides insights and discussions, which can help researchers, practitioners, and stakeholders develop more effective occupancy detection solutions for smart buildings.

## 1. Introduction

Smart buildings provide a productive and cost-effective environment by optimizing their four basic components (structure, system, services, and management) and the interrelationships among them [1]. According to Research and Markets, the global smart building market is expected to reach USD 120.6 billion by 2026, growing at a Compound Annual Growth Rate (CAGR) of 10.6% from 2021 to 2026 [2]. To enhance various building functions, including lighting, Heating, Ventilation, Air Conditioning (HVAC), security, and energy management, these smart buildings use various technologies, including sensors, automation systems, artificial intelligence, and the Internet of Things (IoT). Smart buildings can provide many benefits, including reduced operating costs, improved occupant comfort and safety, and enhanced sustainability. Additionally, using smart technology in buildings can improve the overall management of building systems, making it easier for building managers to monitor and control building functions remotely.

The Amsterdam Edge building is a prime illustration of a smart structure [3]. According to the source above, the Edge is a 430,000-square-foot structure with over 28,000 sensors to collect data on occupancy, temperature, light, humidity, and movement [3]. The sensor data control the building’s systems, such as heating and lighting, to optimize energy consumption and create a comfortable and healthy work environment. The Edge also uses a smart system to manage the parking lot, allowing employees to reserve a parking space through an app, reducing traffic and the time spent searching for a parking spot. The building also includes a smartphone app allowing employees to adjust their workspace environments, such as lighting and temperature, to their preferences. The Edge has achieved the highest sustainability rating possible and is a prime example of how smart buildings can improve occupant comfort, energy efficiency, and overall building performance [3].

Smart buildings can adapt to changing conditions and user preferences using Machine Learning (ML) algorithms and predictive analytics. Significant energy savings and improved occupant comfort can be achieved by optimizing building operations based on real-time data and user behavior patterns. In addition, smart buildings can use data analytics to identify areas of inefficiency and provide recommendations for improvements. This includes upgrading to more energy-efficient systems or adjusting the building’s design to optimize natural light and ventilation [4,5].

Besides the benefits to individual structures, smart buildings can have broader societal benefits by reducing carbon emissions and improving sustainability. Buildings consume a considerable part of the world’s energy, and the widespread adoption of smart building technologies can have a significant impact [5]. Furthermore, the ability of smart buildings to collect and analyze data on energy use and occupant behavior can provide valuable insights for urban planners and policymakers. They could use this information to make better decisions about building codes and infrastructure investments. Overall, the potential benefits of smart buildings are numerous and varied, making them an important area of focus for the building industry and beyond.

An IoT network is a collection of physical devices or “things” connected to the internet and equipped with electronics, software, sensors, and network connectivity [6]. This technology is revolutionizing how people interact with their environment, making consumers’ daily lives easier. IoT technology has created a need for intelligent, autonomous gadgets, allowing for automation and remote control of processes. With the introduction of IoT technology, physical objects can now be embedded with electronics and sensors, enabling them to interact with their environment in many ways [6].

Smart IoT devices have revolutionized the concept of smart buildings. These devices use sensors and embedded systems to gather and analyze data, providing real-time insights into various building systems, including lighting, HVAC, and security [4]. Building managers can cut operating expenses, increase tenant comfort, and optimize energy use with these devices [7,8]. According to a research paper by Statista [9,10], the global market size of smart building technology is expected to reach USD 157.1 billion by 2026, with a CAGR of 16.8%. Another research paper by MarketsandMarkets states that the smart building IoT market is expected to grow from USD 7.4 billion in 2020 to USD 22.2 billion by 2025, with a CAGR of 24.9% [9,10].

Smart IoT devices in smart buildings are also contributing to sustainability efforts. IoT-based smart device automation systems can reduce energy usage in commercial buildings by up to 30%. Furthermore, these systems can reduce greenhouse gas emissions and contribute to achieving environmental sustainability goals [11]. Numerous home, workplace, and healthcare applications have been made possible by using smart devices that do not need human interaction [12]. In the industrial sector, the automation of processes can result in more efficient production and improved safety standards [12]. For example, monitoring systems can be set up to detect hazardous conditions and alert the responsible personnel. Using smart devices without human intervention can provide more accurate and efficient medical treatments in healthcare [12]. Automated monitoring systems can detect irregularities in vital signs and alert the responsible personnel, allowing for the early diagnosis and treatment of illnesses.

It is also advantageous economically to use smart devices without human interaction. Process automation can decrease labor costs as fewer personnel are needed to operate the devices [12]. This can result in an increase in profitability for businesses, as well as a decrease in operational costs. Additionally, the automation of processes can decrease the time needed for production or services, allowing for the completion of tasks much faster than before [6]. This can result in increased customer satisfaction and improved customer loyalty.

The use of smart devices without human intervention has its drawbacks. The automation of processes can lead to a decrease in jobs, as fewer personnel are needed to operate the devices [12]. Additionally, using smart devices can decrease the quality of the services or products, as the devices cannot consider human factors like emotion and intuition [6]. Additionally, using these smart devices without human intervention can lead to a lack of accountability and responsibility, as the devices can malfunction or be hacked, leading to serious consequences [12].

The ability to cut off superfluous lights and HVAC systems when not in use makes occupancy detection a key component of smart building systems. Since buildings use a lot of energy and resources, the goal is to eliminate energy waste in vacant places [13]. There are several ways to achieve occupancy detection, including using sensors such as infrared, ultrasonic, video cameras, etc. These sensors can be used to control lighting, HVAC systems, and security systems while preserving the privacy of occupants [6]. Smart occupancy detection devices are designed to be energy-efficient and provide accurate readings.

Occupancy detection devices can detect occupants’ presence and adjust the lighting, temperature, and ventilation accordingly. Occupancy-based lighting control can save up to 45% of lighting energy consumption in commercial buildings. Occupancy-based HVAC control can save up to 30% of energy consumption in commercial buildings [14]. Moreover, occupancy detection can provide valuable insights into occupants’ behavior, improving occupant comfort and energy efficiency and identifying areas of improvement in the building’s design [15]. The information gathered from occupancy detection can be utilized to locate places with insufficient lighting or uncomfortable temperatures. This information can then adjust the systems accordingly [16].

In addition to energy savings, occupancy detection can help improve air quality and HVAC performance. By detecting occupancy in real-time, building systems can adjust ventilation rates and airflow to maintain optimal indoor air quality, reducing the risk of indoor air pollution and other health hazards. Similarly, by ensuring HVAC systems are only activated when necessary and modifying system parameters in response to real-time occupancy data, occupancy detection can help HVAC systems operate more efficiently. This can help reduce wear and tear on HVAC equipment, extend lifespan, and reduce maintenance costs over time [4].

Although occupancy detection is important and has been studied by many researchers, there is no comprehensive survey of these technologies. While the existing surveys have provided valuable insights into occupancy detection technologies, they are limited in their coverage of specific algorithms or methods. Furthermore, many of these surveys lack a comprehensive analysis of the challenges and limitations of these technologies in real-world deployment and do not provide sufficient insights into future research directions. Therefore, in this paper, we provide a comprehensive survey focusing on the challenges and opportunities in occupancy detection and comparing the various techniques used for occupancy detection. We aim to fill the gap in the existing literature and provide a valuable resource for researchers. The main objective of this research is to identify existing occupancy detection algorithms and sensors for smart buildings using IoT sensors, analyze the advantages and disadvantages of each method, and identify the most suitable method for occupancy detection in smart buildings, depending on the application requirements. Furthermore, this research aims to identify the challenges associated with occupancy detection in smart buildings, such as noise and interference, privacy and security issues, and the need for robust algorithms. Our contributions are highlighted as follows:

- Our paper provides a comprehensive overview of state-of-the-art sensor devices, occupancy detection methods, and detection architecture. We summarize their advantages and limitations. We also classify the occupancy detection methods into traditional methods and machine learning methods.
- Our paper compares the performance of different occupancy detection methods and provides recommendations for their optimal usage in different building environments.
- Our paper analyzes the challenges in real-world deployment and provides insights into future research directions. Identifying potential applications of occupancy detection beyond energy efficiency, such as improving indoor air quality and enhancing building security.

**Figure 1:**
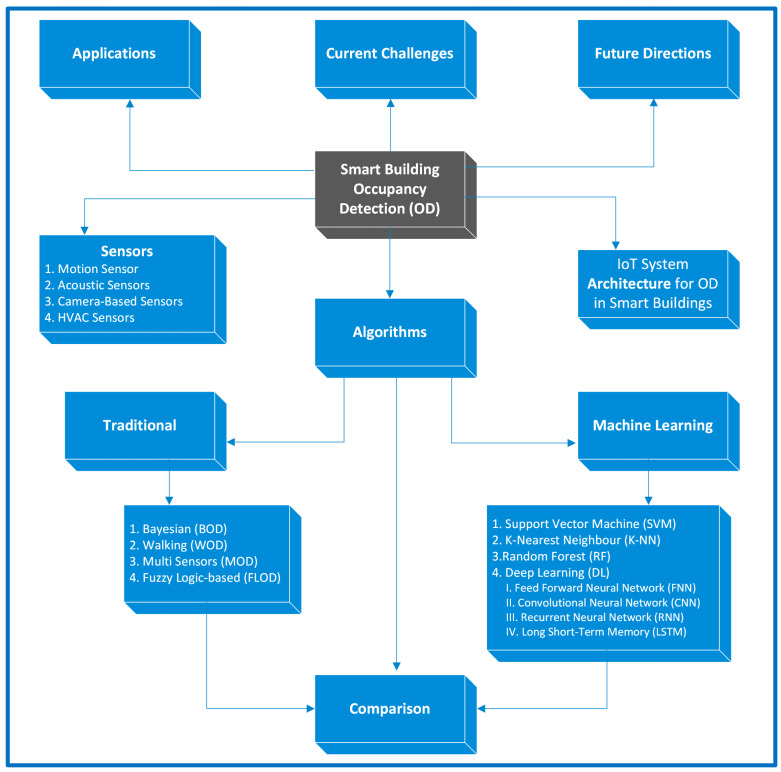
The structure of the paper outlines the detection of occupancy within smart buildings.

Figure 1 illustrates the structure of the paper in this review to categorize existing studies based on various factors, including sensors, algorithms, comparisons, IoT architecture, applications, current challenges, and future directions.

The subsequent sections of the paper are organized as follows: We elaborate on the review methodology of the paper in Section 2. Section 3 discusses the various sensors used for occupancy detection and comparison of the sensors. The architecture of IoT sensors for occupancy detection and smart building occupancy detection is described in Section 4. Section 5 discusses traditional and machine learning occupancy detection algorithms and their comparison. In Section 6, we discuss some scenarios of occupancy detection. Section 7 represents challenges and future work. Finally, we conclude the paper in Section 8.

## 2. Review Methodology

We initially conducted an extensive literature search across various databases, namely MDPI, Elsevier, ACM, and IEEE. We focused on works addressing specific aspects such as occupancy detection, sensors, data collection, IoT, traditional and machine learning algorithms, building type, performance, and limitations. We employed diverse combinations of keywords and their synonyms during our search. Consequently, this review encompasses research studies published between January 2014 and December 2023, chosen to assess recent and relevant contributions while ensuring an adequate number of studies for a thorough examination. The literature reviewed comprises English-written, peer-reviewed journal articles, conference proceedings papers, and book chapters.

### 2.1. Study Selection

The selection process employed in this review adheres to the specifications of the Web of Science, which serves as both a research tool supporting a wide range of scientific tasks across various knowledge domains and a dataset for large-scale data-intensive studies. Specifically, a search was conducted for the past decade (January 2014–December 2023) to focus exclusively on the latest trends in occupancy sensors and traditional ML/DL algorithms for occupancy detection while ensuring a sufficient number of studies for discussion. Duplicate references were eliminated using reference manager software, and only the remaining frameworks were considered after filtering based on their titles, keywords, and abstracts.

### 2.2. Inclusion/Exclusion Criteria

All selected frameworks were thoroughly screened and reviewed based on the inclusion/exclusion procedure outlined below: (1) Frameworks considering different algorithms for occupancy detection in smart buildings were examined. (2) Only studies published between January 2014 and December 2023 were investigated. Research publications accessible online (i.e., peer-reviewed conference proceedings papers, book chapters, and journal articles) were included. (3) In cases where the same authors published multiple frameworks addressing the same problem, the most recent and valuable ones were analyzed. Table 1 compares survey research conducted and published from 2014 to 2023 and our work. Our work provides a more comprehensive study of the related topics.

## 3. Sensors for Occupancy Detection

In this section, we first introduce some major sensors used for occupancy detection and then compare them in the following subsections.

### 3.1. Occupancy Detection Sensors

Occupancy detection sensors can determine the presence or absence of individuals in a specific area. They are a crucial part of building automation systems and are widely employed in numerous applications, including lighting, HVAC, and security [6]. This subsection of the literature review will focus on different occupancy sensors, their advantages, and disadvantages, and how they are used in modern smart buildings.

#### 3.1.1. Motion Sensors

Motion sensors are devices designed to detect movement in their surroundings [28]. They are frequently utilized in smart buildings to automate certain functions and increase the building’s overall efficiency [6]. Motion sensors in smart buildings have the primary benefit of reducing energy usage by turning off lights and other appliances when they are not in use. Motion sensors can automatically turn off lights and other equipment when a room is empty, conserving energy and cutting costs [6]. In addition, motion sensors can also improve security by detecting when someone enters a restricted area or if there is an unexpected movement during non-business hours, alerting the security team in real-time [6]. Overall, motion sensors play a critical role in creating smart and sustainable buildings, helping to improve efficiency and security and reduce energy costs.

#### 3.1.2. Acoustic Sensors

Acoustic sensors are devices that can detect sound waves and vibrations in their environment [29]. In smart buildings, acoustic sensors monitor and analyze various sounds, including voice, music, and ambient noise. The main advantage of acoustic sensors in smart buildings is their ability to improve the overall experience for occupants by controlling and optimizing sound levels [29]. Acoustic sensors can be used to monitor noise levels in common areas such as lobbies, hallways, and conference rooms and automatically adjust sound levels to ensure that they are appropriate for the space and the activities. Additionally, these sensors can help to detect and locate sources of unwanted noise, such as mechanical equipment or construction work, and provide real-time alerts to building managers, allowing them to take corrective action quickly [30]. By controlling and optimizing sound levels, acoustic sensors can help to create a more comfortable and productive environment for building occupants, leading to increased satisfaction and productivity.

#### 3.1.3. Camera-Based Sensors

Camera-based sensors are one of the most crucial components of smart building technologies. These sensors utilize advanced imaging technology to detect and track motion in and around a building [31]. The main advantage of camera-based sensors in smart buildings is their ability to provide real-time surveillance and monitoring of the building’s occupants and surrounding areas. This technology can be utilized for several things, like tracking movement and occupancy trends and security and safety monitoring [31]. Camera-based sensors can also be used to analyze foot traffic patterns, which can help building managers optimize the use of space and improve the overall flow of people throughout the building. Additionally, these sensors can help to reduce energy consumption by adjusting lighting and temperature settings based on occupancy patterns [31]. Overall, camera-based sensors are an important component of smart buildings, providing real-time data and analytics that can be used to optimize building performance and enhance the overall occupant experience.

Motion/acoustic sensors are used in some applications with camera-based sensors. First, camera-based sensors are turned on when a motion sensor detects motion or an acoustic sensor detects sound. Camera-based sensors are turned off if there is motion or acoustic sound for a time period beyond a threshold. This can save energy and reduce useless data recorded.

#### 3.1.4. HVAC Sensors

HVAC sensors are a critical component of smart building technology that helps to observe and control HVAC systems [4]. The main advantage of HVAC sensors in smart buildings is their ability to optimize energy consumption and reduce costs while maintaining comfortable indoor temperatures. These sensors can detect temperature, humidity, and air quality changes and adjust the HVAC system settings accordingly [4,14]. For example, When a room is empty, HVAC sensors may adjust the temperature to save electricity. They can also monitor outdoor weather conditions and adjust the HVAC system to maintain optimal indoor conditions, reducing the workload on the system and extending its lifespan. In addition, HVAC sensors can help to improve indoor air quality by monitoring carbon dioxide levels, humidity, and pollutants and adjusting the HVAC system accordingly [4,14]. Overall, HVAC sensors are critical in creating smart, energy-efficient buildings that give inhabitants a cozy and healthy atmosphere while reducing costs and improving sustainability.

#### 3.1.5. Communication as Sensing

Recent years, there has been a lot research work on the integration of communication and sensing [32]. Next, we discuss WiFi sensing and Bluetooth sensing. WiFi and Bluetooth are communication-as-sensing technologies and play pivotal roles in occupancy detection within smart buildings. This approach utilizes the inherent communication signals emitted by WiFi and Bluetooth-enabled devices to gather valuable data on occupancy patterns, movement, and behavior within a building environment.

WiFi Sensing: WiFi sensing relies on the signals emitted by WiFi-enabled devices, such as smartphones, laptops, and IoT devices. These devices continually emit signals, and WiFi sensors strategically placed throughout the building capture and analyze these signals. By assessing signal strength, frequency, and other parameters, the system can infer the presence, location, and even the number of occupants within a given space and track their movements over time [33]. This approach provides a non-intrusive means of occupancy detection and high accuracy and coverage, making it well-suited for large-scale occupancy detection in smart buildings [34,35].

Bluetooth Sensing, specifically Bluetooth Low Energy (BLE): Bluetooth sensing, particularly BLE, offers a low-power alternative for occupancy detection. Devices like smartphones and wearables emit periodic signals detectable by BLE sensors that can be detected and used to determine occupants’ presence, location, and movement within a building [34,35]. This technology is especially advantageous for its minimal impact on device battery life and ability to integrate into various IoT devices seamlessly.

### 3.2. Comparison of Occupancy Detection Sensors

To Compare occupancy detection sensors, we have used the following terms.

- Sensor Type: Occupancy detection sensors detect human presence in space. There are different types of sensors available, such as ultrasonic sensors, Passive Infrared (PIR) sensors, and microwave sensors;
- Major Analytical Method: The primary analytical technique used by occupancy detection sensors is identifying environmental changes brought on by human presence. To determine whether a person is in space, the sensors take measurements of several environmental elements like temperature, sound, light, and motion;
- Intrusiveness Level: This refers to how invasive or disruptive the technology or methods used to detect occupancy are to the occupants’ privacy and daily activities. The goal is to strike a balance between effectively monitoring and managing building occupancy while respecting individuals’ privacy;
- Sensor Fusion: Data from several sensors are combined through sensor fusion to increase accuracy and decrease false positives. Sensor fusion is a technique that occupancy detection sensors can utilize to merge data from many sensor types, such as PIR, ultrasonic, and microwave sensors, to increase accuracy;
- Accuracy: Accuracy refers to the sensor’s ability to correctly detect a person’s presence or absence in space. Higher accuracy means fewer false positives and false negatives;
- Occupancy Resolution: Occupancy resolution refers to the level of detail at which the sensor can detect occupancy. For example, some sensors can detect the presence of a person but cannot distinguish between one or multiple people;
- Performance Measures: Accuracy, false positive and false negative rates, response time, and power consumption are performance indicators for occupancy detecting sensors. These metrics can be used to assess the potency and usefulness of various sensors and sensor assemblages.

Table 2 is a comparison chart providing an overview of the sensor fusion, accuracy, occupancy resolution, performance measures major analytical methods for four types of occupancy detection sensors: motion sensors, camera-based sensors, acoustic sensors, and HVAC sensors [29]. Analytical methods such as Passive Infrared (PIR) sensors [36] are commonly used in smart building applications because they are inexpensive and reliable. They detect changes in infrared radiation and are typically installed on ceilings or walls. When motion is detected, the sensors signal the building control system to adjust the lighting, HVAC, or other equipment as needed [6,14,37].

Ultrasonic sensors emit high-frequency sound waves and measure the reflection of these waves to detect motion. They frequently work with PIR sensors to increase precision and limit false alerts. Ultrasonic sensors are particularly useful in areas where PIR sensors may not be effective, such as open-plan offices or areas with obstructions [6,14,37,38].

Low-power microwaves are produced by microwave sensors, which then track the waves’ reflections to find movement. They are particularly useful in areas where PIR and ultrasonic sensors may not be effective, such as outdoor areas or areas with extreme temperature variations. Microwave sensors are often combined with PIR or ultrasonic sensors to improve accuracy and reduce false alarms [6,14,37].

A combination of PIR, ultrasonic, and microwave sensors can be used in smart buildings to provide comprehensive and accurate occupancy detection. These sensors can work together to provide redundancy and minimize false alarms. They can also be used to provide more granular data on occupancy patterns and help building managers optimize energy usage and comfort levels.

Motion sensors, such as PIR and ultrasonic sensors, detect motion through environmental changes, such as infrared radiation or ultrasonic waves. They have high accuracy for detecting motion but cannot distinguish between humans and other moving objects.

Camera-based sensors use cameras to capture and analyze visual data to detect human presence. They can detect human presence accurately and distinguish between humans and other objects. However, they require high image quality and processing speed, which can be costly and time-consuming.

Acoustic sensors detect human presence through sound, such as decibel levels or frequency changes. They are accurate and can distinguish between humans and other sound sources.

HVAC sensors detect human presence through changes in temperature or carbon dioxide levels. They have low to moderate accuracy in detecting human presence and cannot distinguish between humans and other temperature or carbon dioxide change sources.

Considering the space’s unique requirements is crucial when choosing the optimum sensor type for a certain application. For example, if the space has high ceilings or large open areas, ultrasonic sensors may be more suitable for detecting motion. Acoustic sensors may be more effective if the space has a lot of background noise, such as in a busy office. Data from many types of sensors can be combined through sensor fusion to increase accuracy and minimize false positives. For example, camera-based sensors can be combined with PIR sensors to improve accuracy in detecting human presence. Infrared radiation changes can be detected using PIR sensors, while visual confirmation of human presence can be obtained via camera-based sensors.

Overall, Table 2 is a comparison chart of four types of occupancy detection sensors: motion, camera-based, acoustic, and HVAC sensors. It outlines their analytical methods, accuracy, occupancy resolution, and performance measures. It is suggested that using sensor fusion will increase precision and decrease false positives. Based on the particular needs of the space and the desired use case, the appropriate sensor type for a certain application can be chosen.

### 3.3. Usage of Sensors in Smart Buildings

Table 3 compares the usage of four different types of sensors—motion sensors, camera-based sensors, acoustic sensors, and HVAC sensors—in residential and commercial buildings. We have used the following terms to compare the use of occupancy sensors in smart buildings.

- Type of Sensor: This column lists the different types of sensors commonly used in buildings. Each sensor type has unique features and functions that suit specific applications.
- Building Type: This column specifies the type of building where the sensors are commonly used. Motion and acoustic sensors are used in both commercial and residential buildings, while camera-based and HVAC sensors are primarily used in commercial buildings.
- Application System: This column lists the specific application system for commonly used sensors. Motion sensors are used for lighting control, camera-based sensors for security and surveillance, acoustic sensors for occupancy detection, and HVAC sensors for temperature and humidity control.
- Centralized/Decentralized: This column specifies whether the energy savings associated with the sensor are centralized or decentralized. Centralized energy savings refer to situations where the sensors are connected to a central control system that manages the entire building’s energy usage. Decentralized energy savings refer to situations where each sensor manages energy usage in a specific area or room.
- Energy Saved: This column lists the approximate energy savings associated with each sensor type. These savings are based on research and case studies conducted in various types of buildings.
- Cost: This column lists the approximate cost of implementing each sensor type in a building. The cost varies depending on factors such as the size of the building, the number of sensors required, and the complexity of the application.

The motion sensor is the first category of sensors listed. Both residential and commercial buildings frequently utilize motion sensors to regulate lights. These sensors can detect motion in a space and control the lighting accordingly. Up to 30% more energy can be saved as a result of this. Decentralized deployment is the norm for motion sensors, which means they are positioned in specific rooms or areas. Motion sensor installation is reasonably inexpensive.

The second type of sensor listed is a camera-based sensor. These sensors are primarily used in commercial buildings for security and surveillance. They are centralized and can monitor the entire building, reducing the need for on-site security personnel. The energy savings associated with camera-based sensors are high due to the increased surveillance efficiency, but the implementation cost is typically high.

Acoustic sensors are the third category of sensors mentioned. Acoustic sensors are utilized in both residential and commercial structures to detect occupancy. These sensors can pick up sound waves and assess whether or not a room is occupied. Energy savings of up to 20% can be achieved by adjusting lighting, HVAC systems, and other equipment with the help of this information. The deployment of acoustic sensors is often dispersed at a moderate cost.

The fourth and final type of sensor listed is the HVAC sensor. These sensors are used for temperature and humidity control in commercial buildings. They can measure the temperature and humidity in a room and adjust the HVAC systems accordingly, leading to energy savings of up to 30%. HVAC sensors are typically deployed in a centralized manner, meaning they are installed throughout the building and controlled by a central system. The cost of implementation is moderate.

The concept of communication as sensing involves repurposing the existing communication infrastructure in smart buildings for occupancy detection. Using WiFi routers and Bluetooth devices, originally designed for communication purposes, as distributed sensors, the building’s network can passively monitor and interpret signals emitted by devices [34,35]. This transformation turns the communication network into an intelligent and unobtrusive occupancy detection system. Smart buildings’ WiFi and Bluetooth sensing technologies encompass various applications, contributing to enhanced efficiency, safety, and user experiences. Some usages include the following:

- Occupancy Detection: Real-time Monitoring:WiFi and Bluetooth sensing technologies enable continuous monitoring of spaces, providing real-time data on occupancy levels, movement patterns, and the utilization of different areas within the building [39,40]. Adaptive Systems: The data collected helps dynamically adapt building systems such as lighting, HVAC, optimizing energy usage based on actual occupancy.
- Energy Management: Plug Load Optimization: WiFi and Bluetooth sensing contribute to efficient plug load energy management by identifying and controlling the usage of energy-consuming devices in occupied spaces, reducing overall energy consumption. Context-Aware Controls: Understanding occupancy patterns allows for context-aware controls, such as adjusting lighting and climate settings based on the specific requirements of each area [39,40,41,42].
- Space Utilization Insights: Resource Allocation: Analyzing communication signals provides insights into popular gathering areas, high-traffic zones, and underutilized spaces. This information aids in optimizing resource allocation and space utilization for improved functionality and user satisfaction [40]. Workspace Design: Understanding how spaces are utilized allows for the design of workspaces that align with actual usage patterns, fostering a more productive and comfortable environment.
- Security and Emergency Response: Occupant Tracking: WiFi and Bluetooth sensing technologies play a vital role in tracking occupant locations during emergencies, ensuring swift and targeted responses for evacuation or assistance [39,41]. Security Monitoring: These sensing technologies contribute to security monitoring by providing information on the movement and presence of individuals within the building, enhancing overall security measures.
- User Experience Enhancement: Personalized Services: Context-aware insights derived from communication signals enable the delivery of personalized services to building occupants, enhancing their overall experience within the smart building environment. Automation and Convenience: By understanding occupancy patterns, smart building systems can automate processes and provide convenient services, such as automated check-ins, room bookings, and tailored environmental settings.
- Maintenance and Facility Management: Predictive Maintenance: Analyzing occupancy data can assist in predicting maintenance needs by identifying areas that experience higher usage and may require more frequent inspections or repairs. Efficient Cleaning Schedules: Knowledge of space utilization patterns aids in optimizing cleaning schedules based on actual demand, contributing to more efficient facility management.
- Compliance and Reporting: Occupancy Reporting: WiFi and Bluetooth sensing technologies facilitate accurate reporting on occupancy levels, helping building managers comply with regulations and guidelines related to occupancy limits and safety standards [40,41].

## 4. IoT System Architecture for Smart Building

In this section, we survey IOT system architecture for occupancy detection.

### 4.1. IoT System Architecture

**Figure 2:**
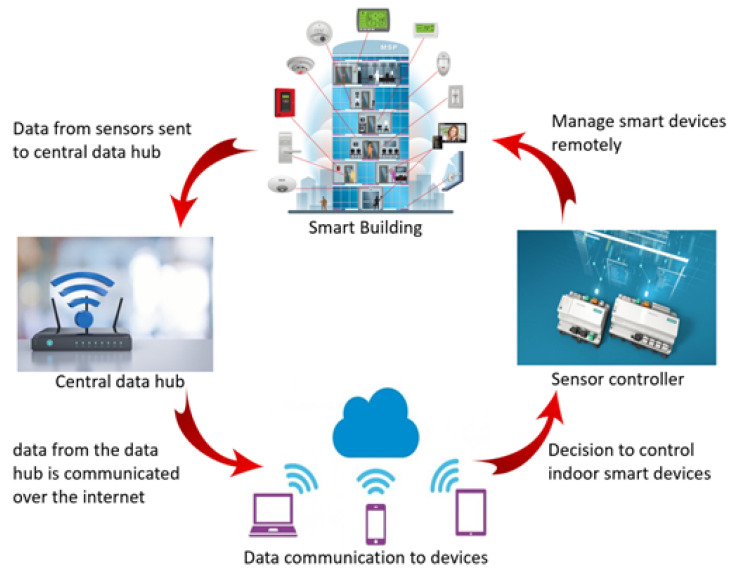
How the occupancy detection for smart buildings using IoT sensors system works [14].

The IoT system architecture for a smart building typically involves collecting data from sensors in the room, sending that data to a data fusion center, communicating that data over the internet, using that data to make decisions about controlling indoor equipment and controlling the smart building system, shown in Figure 2.

- Collecting sensor data: Sensors in the room can detect things like temperature, humidity, light, and occupancy. These data are collected and sent to a data fusion center.
- Data fusion center: The data fusion center is responsible for receiving data from multiple sensors and integrating it into a single, cohesive view of the building. This includes identifying patterns and anomalies in the data that can inform decisions about how to control the building.
- Data communication: The data from the data fusion center are typically communicated over the internet through a wired or wireless connection.
- Decision-making: Based on the data collected and analyzed by the data fusion center, decisions are made about controlling the indoor equipment in the building. For example, if a room is too warm, the HVAC system might be adjusted to decrease the temperature.

### 4.2. Occupancy Detection in Smart Buildings

**Figure 3:**
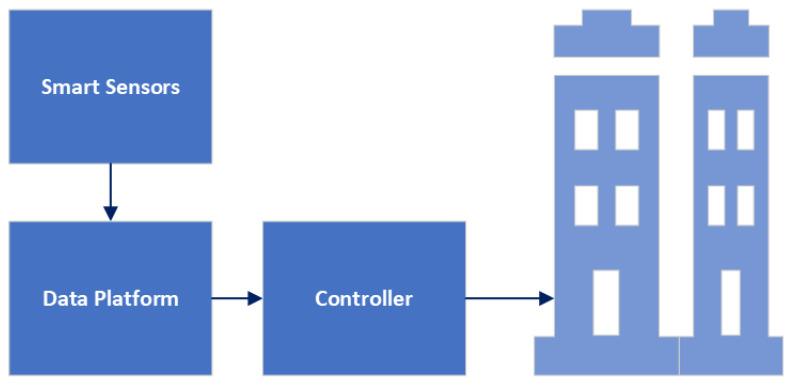
IoT system architecture for occupancy sensing [11].

Figure 3 shows an example of architecture occupancy sensing. In Figure 3, the sensors can detect occupancy through various methods, such as passive infrared (PIR) and active infrared (AIR) detection. PIR sensors detect occupancy by picking up on infrared radiation emitted by occupants in the room. AIR detection utilizes infrared emitters to detect occupancy [14]. These sensors can detect even subtle movements and can be used to detect occupancy in large areas. The data from the sensors are then sent to a data platform, which stores and analyzes the information gathered from the sensors [14]. This data platform can be used to monitor occupancy levels in a building in real-time and detect any anomalies or irregularities in occupancy. The platform can use algorithms to analyze the data and generate insights such as occupancy patterns, peak usage hours, and areas with low utilization.

These data can then send commands to a control system like a smart building. This control system can be used to adjust settings to optimize energy efficiency and control security systems and other systems in the building [14].

Occupancy detection in smart buildings using IoT sensors can offer several benefits, some of which are:

- Energy efficiency: Occupancy detection sensors can help reduce energy consumption by turning off lights, HVAC systems, and other equipment in areas that are not in use. This can significantly reduce energy waste and lower utility bills.
- Improved space utilization: By analyzing occupancy data, building managers can identify underutilized areas and adjust to better utilize space. For example, a conference room that is rarely used can be repurposed as a workspace, helping to optimize the use of building resources.
- Enhanced comfort: Occupancy detection sensors can help to maintain a comfortable environment for occupants by adjusting temperature, lighting, and other environmental factors based on occupancy patterns. This can help to improve the overall occupant experience and productivity.
- Increased security: Occupancy detection sensors can be used to monitor and control access to sensitive areas of the building. This can help to enhance security by preventing unauthorized access.
- Data-driven decision-making: By collecting and analyzing occupancy data, building managers can gain insights into occupancy patterns, peak usage hours, and areas with low utilization. These data can be used to make informed decisions on resource allocation and building operations.
- Cost savings: By optimizing energy usage, improving space utilization, and enhancing comfort, occupancy detection sensors can help lower operating costs and improve the overall financial performance of the building.

Next, we discuss communication technologies, processing cores, data fusion, analytics, control systems, and user interfaces.

- Communication Technologies: Occupancy detection in smart buildings is a multifaceted process that hinges on integrating wired and wireless advanced communication technologies, processing cores, data fusion, control systems, and user interfaces. This comprehensive approach enables the efficient collection, analysis, and utilization of data for optimal building management. Various communication protocols play a crucial role in fostering seamless connectivity across the different components of the system:
  – Wired Communication: [43] Ethernet: Wired Ethernet is a reliable and high-speed communication technology that connects a building’s sensors, devices, and control systems. It ensures robust data transmission and is suitable for applications where stability is crucial.
  – Power Line Communication (PLC): PLC utilizes existing electrical wiring for data transmission. This wired communication method is beneficial in scenarios where additional wiring may be challenging, providing an alternative connectivity means.
  – Serial Communication: Serial communication interfaces, such as RS-485, are employed for connecting devices in a daisy-chain fashion. This is useful in scenarios where multiple sensors need to communicate over longer distances.
  – Wireless Communication: [43]
    * Wi-Fi (IEEE 802.11): Wi-Fi is a fundamental wireless technology in smart buildings, operating at 2.4 GHz and 5 GHz frequency bands [44]. It provides high data rates and reliable connectivity, with strategically placed Wi-Fi access points ensuring comprehensive coverage throughout the building.
    * Zigbee (IEEE 802.15.4): Zigbee is a low-power, low-data-rate wireless protocol designed for sensor networks. Zigbee devices form a mesh network, enabling sensors to communicate and relay data efficiently. Its usage spans smart lighting, temperature control, and occupancy sensing applications.
    * LoRaWAN (long-range wide area network): For long-range communication with low power consumption, LoRaWAN operates in sub-GHz frequency bands (e.g., 868 MHz in Europe and 915 MHz in the US) [45]. LoRaWAN gateways collect data from sensors across a wide area, making it ideal for large-scale deployments in smart buildings.
    * Bluetooth Low Energy (BLE): BLE, energy-efficient and operating over short distances, is employed for device-to-device communication within smart buildings. It is commonly used to connect smartphones, wearables, and beacons [40].
- Processing Cores: Beyond communication protocols, the efficacy of occupancy detection also relies on sophisticated processing cores, including microcontrollers (MCUs), system-on-chip (SoC) solutions, and edge servers [42]:
  – Microcontrollers (MCUs): MCUs, integrated systems with processors, memory, and peripherals, serve as the backbone of IoT devices. Cost-effective and widely used in sensor nodes, MCUs handle sensor data, execute algorithms, and manage power efficiently.
  – System-on-Chip (SoC): SoCs integrate multiple components into a single chip, including CPU, memory, and radio. Efficient for edge devices and sensor nodes, SoCs contribute to compact, energy-efficient designs.
  – Edge Servers: In larger buildings, edge servers locally process data before transmitting it to the cloud. These servers handle complex analytics, equipped with powerful processors, such as ARM-based architectures, ensuring faster response times.
- Data Fusion and Analytics: In tandem with communication technologies and processing cores, data fusion and analytics play a critical role:
  – Data Fusion Center: This central hub aggregates data from various sensors, employing techniques like Kalman filtering or Bayesian inference to enhance data accuracy. It combines information from temperature sensors, motion detectors, and other sources.
  – Analytics Algorithms: Machine learning algorithms analyze sensor data for various purposes, including occupancy prediction and anomaly detection. Predictive models estimate future occupancy levels, while anomaly detection algorithms identify irregularities that may indicate security breaches or equipment malfunctions.
- Control Systems: The culmination of these components facilitates effective control systems and user interfaces:
  – Building Management System (BMS): The BMS monitors and controls building equipment based on real-time occupancy information. It receives sensor data and adjusts HVAC, lighting, and access control system settings [46].
  – Actuators: Actuators, such as motorized blinds and smart thermostats, respond to control signals triggered by occupancy data. For instance, lighting levels or room temperatures are adjusted when occupancy is detected.
- User Interface:
  – Dashboard: Building managers and occupants interact with the system through a web-based or mobile dashboard. Real-time occupancy insights, energy usage, and alerts are displayed for efficient monitoring [46].
  – Mobile Apps: Occupants can use mobile apps to adjust settings like lighting and temperature based on their preferences. For instance, employees can book meeting rooms through an app, considering real-time occupancy availability.
- Privacy Considerations:
  – Anonymization: Occupancy data is anonymized to protect individual privacy, avoiding associations with specific individuals.
  – Consent: Occupants’ consent is obtained regarding data collection and usage, transparently communicating how occupancy data will be utilized.

The interplay of communication technologies, processing cores, data fusion, analytics, control systems, and user interfaces creates an intelligent, responsive, and efficient smart building ecosystem. This approach optimizes energy usage, enhances comfort, and ensures sustainable resource utilization while privacy considerations safeguard individual rights.

## 5. Occupancy Detection Algorithms

We classify occupancy detection algorithms into two broad categories: machine learning and traditional algorithms. Traditional algorithms are rule-based and rely on pre-defined rules and thresholds to detect occupancy. However, machine learning algorithms use complex statistical models and mathematical illustrations of observed data that assist analysts and data scientists ro see connections and patterns within datasets. They can be used to generate sample data and make real-world predictions [47] and algorithms to learn and adapt to changing occupancy patterns. We survey them in the following two subsections.

### 5.1. Traditional Occupancy Detection Algorithms

These algorithms typically use simple sensors such as motion sensors, door contacts, or infrared sensors to detect environmental movement or changes. They can be effective in simple environments with consistent occupancy patterns but may struggle to adapt to complex or dynamic environments with varying occupancy patterns. We survey some of the major methods as follows.

#### 5.1.1. Bayesian Occupancy Detection (BOD) Algorithm

In probability theory, Bayes’ rule, often known as Bayes’ theorem or Bayes’ law, is a key theorem that explains how to update the probability of an occurrence in light of new evidence or new data [48]. According to Bayes’ rule, which is expressed mathematically, the likelihood of the evidence given the hypothesis P(E|H) is proportional to the product of the marginal likelihood of the evidence P(E) and the prior probability of the evidence P(H):

(1) P(H|E)=P(E|H)∗P(H)/P(E)

where P(H|E) is the posterior probability of the hypothesis given the evidence, which is what we want to compute; P(E|H) is the likelihood of the evidence given the hypothesis, P(E|H) is the prior probability of the hypothesis before observation of the evidence, and P(E) is the marginal likelihood of the evidence, which is the likelihood of observing the evidence given all conceivable hypotheses [30,49,50].

**Figure 4:**
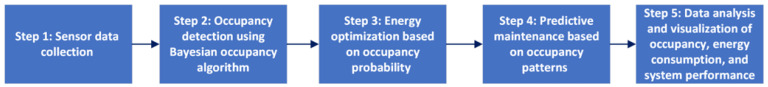
Occupancy detection using the BOD Algorithm.

The Bayes’ rule offers a method for updating a hypothesis’ probability when new information or evidence becomes available. It is widely utilized in many disciplines, including data analysis, machine learning, and artificial intelligence [49]. The Bayesian occupancy detection algorithm can be used in smart buildings to detect the occupancy state of rooms, floors, or the entire building and to optimize energy consumption, ventilation, and other building services accordingly [5,51]. Normally, the method has several steps, as shown in Figure 4 [23,26,30].

Step 1: Sensor Data Collection: Gathering sensor data from the building is the initial stage in using the Bayesian occupancy detection algorithm. Sensors can be deployed throughout the building to collect data on occupancy, temperature, humidity, light, and other parameters relevant to the building’s operation.

Step 2: Occupancy Detection: The occupancy state of the building can be detected by applying the Bayesian occupancy detection algorithm to the sensor data collected. Based on the sensor data, the algorithm calculates the building’s occupancy probability and updates the occupancy probability distribution as new data is collected.

Step 3: Energy Optimization: Once the occupancy state of the building is detected, the algorithm can be used to optimize energy consumption and other building services. For example, if the occupancy probability is low, the algorithm can adjust the ventilation, heating, and lighting systems to save energy [49]. On the other hand, if the occupancy probability is high, the algorithm can increase the ventilation, adjust the temperature, and turn on additional lighting to ensure occupant comfort [48].

Step 4: Predictive Maintenance: The Bayesian occupancy detection algorithm can also be used for predictive maintenance of the building’s systems. By monitoring the occupancy state of the building, the algorithm can predict when building services will need maintenance or replacement, such as when occupancy patterns change or when the building is used more frequently.

Step 5: Data Analysis and Visualization. Finally, insights into the building’s occupancy patterns, energy usage, and system performance can be gained by analyzing and visualizing the data obtained from the sensors. Such data for building managers can assist the decision-making process for maximizing building services and enhancing occupant comfort [5].

The Bayesian occupancy detection algorithm can play a key role in making smart buildings more energy-efficient, comfortable, and cost-effective while enabling predictive maintenance and data-driven decision-making [30,52].

In occupancy detection, complexity arises from sensor fusion, information gain calculation, and probabilistic modeling. Integrating data from diverse sensors requires precise calibration and synchronization. Determining the most relevant measurements involves assessing information gains. Constructing Bayesian networks to model human behavior adds further complexity by establishing cause-and-effect relations. These factors collectively contribute to the computational demands of occupancy detection systems.There are some advantages, disadvantages, and applications of the BOD algorithm in smart buildings explained in Table 4 [5,30,48,49,52].

#### 5.1.2. Walking Occupancy Detection (WOD) Algorithm

The Walking Occupancy Detection (WOD) algorithm is a type of occupancy detection algorithm that uses motion sensors to detect the occupancy of people in a smart building. It is commonly used in smart buildings to optimize energy usage and provide a comfortable environment for building occupants [53,54].

The WOD algorithm uses motion sensors to detect the movement of people in the building. The algorithm identifies a person’s walking speed and determines whether they are entering or leaving a space based on the direction of their movement [53,54]. By analyzing people’s movement patterns in the building, the algorithm can update the occupancy state by the estimated number of persons in each space.

To provide a more detailed explanation of the WOD algorithm, let us look at each step in more detail:

Step 1: Data collection: Motion sensors are installed in different areas of the building to collect data on the movement of people [53,54]. The sensors may be infrared, ultrasonic, or microwave sensors, depending on the building’s layout and requirements. The sensors are typically installed in areas with high foot traffic, such as entrances, hallways, and conference rooms.

Step 2: Motion detection: The WOD algorithm uses a motion detection algorithm to identify the presence of people in each room/space. The algorithm analyzes the motion sensors’ data to detect patterns consistent with human movement. For example, the algorithm may detect a sudden change in the amount of infrared radiation or sound waves in space, indicating the presence of a person [55].

Step 3: Walking speed estimation: Once the presence of people is detected, the algorithm estimates the walking speed of each person based on their movement patterns. This is done by analyzing the time it takes for a person to move from one sensor to another. The walking speed is an important factor in predicting how many people are in each space, as people walking quickly may be counted as multiple people.

Step 4: Direction detection: The algorithm determines the direction of movement of each person to determine whether they are entering or leaving a space. This is done by analyzing the sequence of activated sensors as a person moves through the building. The algorithm can determine whether a person is moving towards or away from a space by analyzing the order in which sensors are activated.

Step 5: Occupancy estimation: The WOD method estimates the number of individuals present in each place using each person’s walking speed and direction. This is carried out by looking at how people move around and calculating how many there are depending on how fast they walk and stay in each area. The algorithm may additionally include data from other sensors, such as temperature and humidity sensors, to increase the accuracy of the occupancy estimation.

Step 6: Occupancy state update: Finally, the occupancy state of each space is updated based on the occupancy estimation. The occupancy state may be represented as a binary variable (occupied/unoccupied) or a continuous variable (number of people present). Utilizing this data will allow for optimizing the building’s energy use and making necessary adjustments to the HVAC and lighting systems. For instance, energy can be saved by turning off or lowering the HVAC and lighting systems when a space is empty.

The WOD algorithm’s overall process entails gathering data from motion sensors, detecting the presence of people in each space, estimating their walking speed, and using this data to estimate the number of people present in each space and update the occupancy state appropriately [53,54]. By modifying HVAC and lighting systems according to the occupancy condition of each space, the WOD algorithm can be utilized to optimize energy usage in smart buildings. For instance, the HVAC and lighting systems can be turned off or lowered to save energy when a space is unattended. The HVAC and lighting systems can also be changed to create a comfortable environment for building inhabitants when a space is filled [55].

The WOD algorithm is a multi-step, intricate process that can be modified to meet the particular requirements of the building. The algorithm may interface with other building systems to optimize energy usage and offer residents a comfortable environment. It can be trained using previous data to increase the accuracy of the occupancy estimation.

Occupancy detection complexity involves choosing suitable sensors like cameras, LiDAR, and radar tailored to the environment. Addressing stillness scenarios, such as occupants working at desks, adds an additional layer of complexity, requiring careful consideration for accurate detection. There are some advantages, disadvantages, and applications of the WOD algorithm in smart buildings explained in Table 5 [53,54,55].

#### 5.1.3. Multi-Sensor Occupancy Detection (MOD) Algorithm

The Multi-sensor Occupancy Detection (MOD) algorithm is a kind of occupancy detection system that employs numerous sensors, including motion sensors, light sensors, and temperature sensors, to locate humans inside a building. It is often used in smart buildings to maximize energy efficiency and give building inhabitants a comfortable atmosphere [56]. The MOD algorithm obtains data from various sensors and analyzes it to assess the occupancy state of each space in the building. The goal is to use the synergistic approach of various error modes of sensors to complement one another so that the result is more than the sum of their parts [56,57].

The Multi-sensor Occupancy Data-driven Estimation System for Smart Buildings (MODES) is one example of MOD. It uses two different cutting-edge sensing methods documented in the literature (thermal and vibration sensors), which are capable of counting the number of occupants in a given zone. A data-driven optimization approach for sensor fusion is then utilized to combine the two occupancy predictions to provide a better estimate. Additionally, a data-driven occupancy model is combined with this recently updated estimate as input for a particle filter to obtain an even more precise estimate [57].

When combining the two occupancy streams using the Data-driven Optimization-based Weighted Average (DOWA) method as part of the MOD algorithm, the Dempster-Shafer Evidence-Based Combination Rule is employed to determine the best fusion weights between the two data streams [58,59]. The DOWA algorithm is a data-driven optimization technique that combines various occupancy streams using a set of weights. Based on the effectiveness of the occupancy estimation algorithm, the weights are optimized [59]. The goal is to use the synergistic approach of various error modes of sensors to complement one another and produce a result that is more than the sum of its parts. Following are the DOWA steps [58,59,60]:

Step 1: Data collection: Multiple sensors are used to accumulate data on the occupancy state of each space in the building. For example, motion sensors can be used to sense the movement of people in a space, light sensors can be used to detect changes in lighting levels, and temperature sensors can be used to detect temperature changes.

Step 2: Data processing: The collected data from the sensors is processed to identify patterns showing each space’s occupancy state. For example, if a motion sensor detects movement in a space and a light sensor detects an increase in lighting levels, this may indicate that the space is occupied.

Step 3: Occupancy state estimation: The data processing results are used to estimate the occupancy state of each space. This may or may not involve using machine learning algorithms to analyze the sensor data and accurately determine the occupancy state.

Step 4: Occupancy state update: The occupancy state of each space is updated based on the occupancy state estimation. The building’s HVAC and lighting systems can be modified using this information to reduce energy consumption and create a comfortable atmosphere for building occupants.

The MOD algorithm is superior to conventional occupancy detection algorithms in a number of ways. The algorithm can estimate the occupancy state more accurately and eliminate false positives and false negatives by using numerous sensors [57]. The algorithm may also be modified to work with various space types and sensor setups, giving it a flexible option for occupancy identification in smart buildings. The MOD algorithm is an effective technique for smart building occupancy detection that can assist in reducing energy consumption and give building occupants a comfortable atmosphere. The MOD algorithm may give precise occupancy status estimations and minimize energy loss by utilizing many sensors and advanced data processing algorithms [57].

Occupancy detection complexity arises from data fusion, integrating information from sensors like infrared, ultrasonic, and video while managing noise. Calibration challenges, ensuring consistent measurements across sensors, add an additional layer of complexity, requiring careful consideration for accurate and reliable detection. Some advantages, disadvantages, and applications of the MOD algorithm in smart buildings are explained in Table 6 [56,57,58,59,60].

#### 5.1.4. Fuzzy Logic-Based Occupancy Detection (FLOD) Algorithm

Fuzzy Logic-based Occupancy Detection (FLOD) is an algorithm for detecting occupancy in a room using sensors and providing real-time feedback to the user [61,62]. This algorithm has been used in many applications, such as home automation and energy management. FLOD is based on a fuzzy logic approach which uses fuzzy sets to represent the room occupancy state. The algorithm is reliable and accurate in detecting occupancy in a room [61].

Sensor data from devices like motion, temperature, and humidity sensors is used as input by the FLOD algorithm. The employment of these sensors allows for the detection of persons in a space. Afterward, the algorithm analyzes the data and generates an output showing whether humans are in the room. The decision-making process is then carried using the output to implement actions like turning on the lights, modifying the room’s temperature, or alerting the user [61,62].

The FLOD algorithm is based on fuzzy set theory, a mathematical approach that uses fuzzy sets to model real-world systems. It is predicated on the notion that a set of values rather than a single value, can be used to represent the state of a system. Fuzzy logic assesses if a given value is a member of a fuzzy set. This membership is based on the value’s degree of truth or truthfulness in the fuzzy set [61]. The FLOD algorithm uses fuzzy sets to represent room occupancy by dividing the room into several cells. Each cell can have a range of values, such as 0–20, 21–40, and 41–60, representing the cell’s occupancy range. The degree of membership of a given value in the cell is determined using this range of values. If a given value is within the range of 0–20, it is considered a low occupancy cell. If the value is within the range of 21–40, it is considered a medium occupancy cell; if the value is within the range of 41–60, it is considered a high occupancy cell.

The FLOD algorithm then uses each cell’s degree of membership to calculate the room’s occupancy state. It calculates the overall occupancy state by combining the individual cell occupancy states. This procedure, known as fuzzy inference, is utilized to determine the room’s overall occupancy condition [16]. The FLOD algorithm is reliable and accurate in detecting occupancy in a room. It has been used in many applications, such as home automation, energy management, and security systems. It is simple to implement and can be used in various environments. The algorithm is also resilient and capable of coping with environmental changes, such as changes in the number of people present in a space or changes in temperature. The algorithm can also handle noisy data and provide reliable and accurate results [61].

A type of many-valued logic known as fuzzy logic deals with reasoning that is approximate rather than exact [16]. It is based on degrees of truth, which allows for a more flexible approach to problem-solving than binary logic. FLOD algorithm uses fuzzy logic to classify occupancy levels in buildings [62]. The algorithm works by taking inputs from various sensors and using fuzzy logic to infer the level of occupancy in the building.

The problem formulation of the FLOD algorithm consists of three main steps [62]. Firstly, the input variables are identified and classified into three categories: environmental, human, and equipment. Environmental variables include temperature, humidity, light intensity, and other environmental conditions, while human variables are related to activities such as presence or movement in the building [63]. Lighting and air conditioning systems are two examples of equipment variables connected to the building’s hardware. The building’s occupancy level is determined using all of these factors [61,62].

The second stage is to define fuzzy sets, which translate input variables into output variables. This uses a fuzzy inference system, which takes the input variables and outputs a fuzzy set. This fuzzy set is then used to determine the occupancy level of the building. The third step is defining the rules to map the fuzzy sets to the output variable. This is accomplished by developing a set of rules that specify how the input variables should be interpreted to calculate the building’s occupancy level. The problem formulation of the FLOD algorithm is an important step in accurately detecting building occupancy levels. It allows for more accurate occupancy detection by considering multiple input variables and mapping them to a single output variable. The fuzzy inference system allows for a more flexible approach to problem-solving than binary logic, which allows for more accurate occupancy detection [16,61,62].

The FLOD algorithm is a helpful tool for precisely determining building occupancy levels. The three key steps in the algorithm’s problem formulation are establishing the fuzzy sets, identifying the input variables, and specifying the rules for mapping the fuzzy sets to the output variable. Unlike binary logic, fuzzy logic offers a more flexible problem-solving method and precise occupancy detection.

Occupancy detection complexity involves crafting fuzzy rules and membership functions for rule design and managing linguistic variables, such as descriptions like “partially occupied”. Balancing these aspects is crucial for developing effective fuzzy logic models in accurate occupancy detection. There are some advantages, disadvantages, and applications of the MOD algorithm in smart buildings explained in Table 7 [16,61,62].

### 5.2. Machine Learning Occupancy Detection Algorithms

These algorithms often create a model of the room and its occupancy patterns using data from various sensors, including temperature sensors, CO_2_ sensors, light sensors, and sound sensors [64]. In order to increase their accuracy and performance over time, machine learning algorithms can be employed for both supervised and unsupervised learning. They can also be trained on enormous datasets. These algorithms are well-suited to complex environments where occupancy patterns are dynamic and unpredictable, and they can provide more accurate and reliable occupancy detection than traditional algorithms [52].

**Figure 5:**
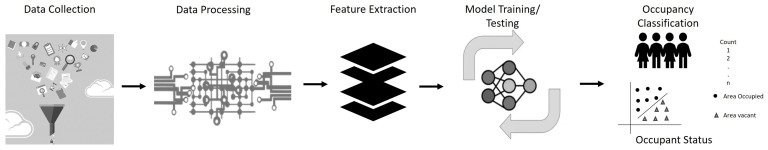
The common steps of the process of occupancy detection via ML/DL.

Occupancy detection using Machine Learning (ML)/Deep Learning (DL) involves several steps, as illustrated in Figure 5. These include collecting data, preprocessing it, extracting features, training and testing models, and categorizing spaces according to occupancy. Each of these steps is important for a successful occupancy detection system [65].

The initial steps of data collection and preprocessing are fundamental and common across most machine learning algorithms, including the four major methods we mentioned: Support Vector Machine, K-Nearest Neighbors (KNN), Random Forest (RF), and Deep Learning (DL). After these initial steps, the algorithms start to diverge in terms of their specific mechanisms for learning and making predictions.

Step 1. Data Collection: To prepare the model for testing and training, this stage entails acquiring data. The information used for occupancy detection would include a number of characteristics or properties about the area or environment being observed. For example, this could include temperature readings, humidity levels, light intensity, CO_2_ concentrations, timestamps, and more [52,66,67].

Data collection might be completed using sensors, detectors, or other data sources capable of capturing relevant information about the environment being monitored. The data should be labeled with the corresponding occupancy status (occupied or unoccupied) to enable supervised learning. The accuracy and representativeness of the data collected directly impact the performance of the trained model. The information should include a range of situations and circumstances the model might face in the real world [52,66].

Step 2. Data Preprocessing: After collecting the data, it must be preprocessed to prepare for training and testing the model. Data preprocessing is a critical step that aims to clean, transform, and organize the data [66,67,68,69]. Common preprocessing steps include the following:

- Handling missing values: If any data are missing, strategies like imputation (filling in missing values) might be used based on the characteristics of the data and the problem.
- Feature scaling/normalization: Features often have different scales, and scaling them to a common range (e.g., between 0 and 1) helps algorithms perform better and converge faster during training.
- Feature selection: Not all features might be relevant or contribute equally to the model’s performance. Feature selection techniques can be employed to choose the most informative features.
- Encoding categorical variables: If the data include categorical variables (e.g., room names), they must be encoded into numerical values for the algorithms to process.
- Splitting the data into training and testing sets: The data are split into two parts: a training set for the model’s training and a testing set for its performance evaluation. Common ratios are 70-30 or 80-20 for training and testing, respectively.
- Handling imbalanced data (if applicable): If one class (e.g., occupied) is significantly more frequent than the other, techniques like oversampling, under-sampling, or generating synthetic samples might be used to balance the dataset.

These first two steps are foundational for all the mentioned algorithms. Data must be collected and preprocessed correctly to guarantee that they are in an appropriate state for each algorithm’s future phases, which will differ based on their unique characteristics and approaches. Next, we introduce several algorithms, including steps 3, 4, 5, 6, and 7.

#### 5.2.1. Support Vector Machine

**Figure 6:**
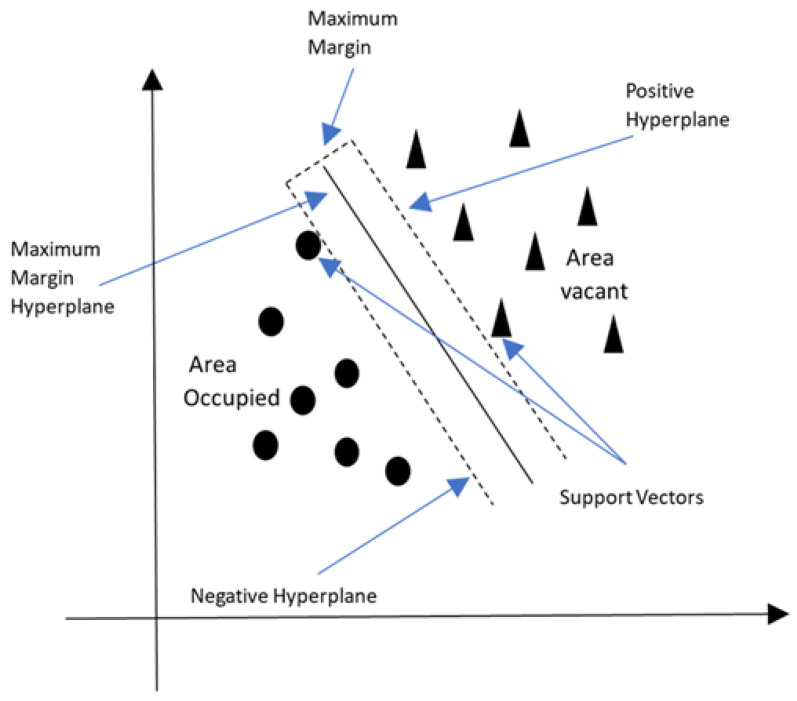
Optimal hyperplane using the SVM algorithm [71].

A supervised machine learning approach called Support Vector Machine (SVM) is utilized for classification and regression tasks [52,64,70]. SVMs can be used in occupancy detection to ascertain if a space (for example, a room) is occupied based on specific traits or qualities. Figure 6 illustrates how SVMs operate by identifying the ideal hyperplane in a high-dimensional feature space that optimally distinguishes between various classes. Figure 6 shows the occupancy classification using the SVM algorithm.

To understand SVM better, next, we introduce its mathematical explanation. Mathematical explanation of SVM: Assume that we have a dataset of occupancy detection with two classes: “Occupied” and “Not Occupied”. Each data point is represented by features, e.g., time of day, temperature, light intensity, etc., [70].

Let *X* denote the set of feature vectors (inputs); Let *Y* denote the corresponding class labels, where Y=+1 for “Occupied” and Y=−1 for “Not Occupied”; Let (Xi,Yi) denote the *i*-th data point and its label. SVM aims to find a hyperplane that maximizes the data points of various classes while preserving a gap between them. The equation represents the hyperplane

(2) w·X+b=0

where *w* is the weight vector perpendicular to the hyperplane, and *b* is the bias term. The distance between a data point *X* and the hyperplane is given by

(3) $$\begin{array}{c} Distance=\frac{\left|w\cdot X+b\right|}{∥w∥} \end{array}$$

Optimal Hyperplane: The ideal hyperplane that optimizes the margin between the two classes is what SVM seeks to identify. The margin separates the two nearest data points to the hyperplane (one from each class) [70]. They are referred to as support vectors. Mathematically, we want to maximize

(4) $$\begin{array}{c} Margin=\frac{2}{∥w∥} \end{array}$$

This can be turned into a minimization problem by minimizing ${\left||w|\right|}^{2}$, subject to the constraint that all data points are correctly classified, i.e., for each data point (Xi,Yi)

(5) $$\begin{array}{cc} Y_{i}\left(w\cdot X_{i}+b\right) & \geq 1 \end{array}$$

This leads to the formulation of the SVM optimization problem

(6) $$\begin{array}{c} \min_{w,b}\frac{1}{2}{∥w∥}^{2} \end{array}$$

(7) $$\begin{array}{cc} \mathrm{Subject}\;\mathrm{to}\;\; & Y_{i}\left(w\cdot X_{i}+b\right)\geq 1\;\mathrm{for}\;\mathrm{all}\;i \end{array}$$

The SVM will learn a hyperplane for occupancy detection that best separates the features associated with occupied and non-occupied rooms. The trained SVM can then classify new feature vectors (feature combinations) into one of the two classes based on which side of the hyperplane they fall.

Step 3. Feature Selection: In this step, we choose which input variables or features to include in the SVM model. The objective is to choose the most pertinent features to classify occupancy status using SVM accurately [52,64,70]. We explain some related terminologies as follows.

- Domain knowledge: Domain experts can provide valuable insights into which features are likely important for occupancy detection. For example, in an office occupancy prediction scenario, features like temperature, humidity, and light intensity might be deemed important
- Feature importance techniques: Machine learning provides various methods to assess the importance of each feature quantitatively. Common techniques include:
  – Correlation analysis: This measures the linear relationship between each feature and the target variable (occupancy status). Features with high absolute correlations are often considered important.
  – Feature importance scores: Algorithms like Random Forest or Gradient Boosting can be used to compute feature importance scores. Features with higher scores are considered more influential.
  – Recursive feature elimination (RFE): RFE retrains the model after iteratively removing the least significant features until the target number of features is obtained.

As choosing irrelevant or redundant features can hurt the SVM performance, the chosen features should be a subset of the original input variables.

Step 4. Model Training: The SVM training process starts after the features are chosen. Finding the hyperplane that best divides the data into various classes is the main goal of SVM. Spaces that are occupied and vacant [52,64,70].

- Hyperplane: The data are divided by this decision boundary. There are two classes in a binary classification problem, such as occupancy detection: occupied and unoccupied. The margin, or the separation between the hyperplane and the closest data points from both classes, should be maximized by the hyperplane.
- Support Vectors: The data points nearest to the hyperplane. They are essential in determining the margin and the hyperplane. These support vectors define the hyperplane.
- Margin: The margin is the difference between the closest support vectors and the hyperplane. Finding the hyperplane that optimizes this margin while reducing classification mistakes is the goal of SVM.

Step 5. Kernel Trick (if necessary): The data may not always be linearly separable in the first feature space. SVM uses a kernel trick to translate the data into a higher-dimensional space where it might become linearly separable in order to handle this. Typical kernel operations include [52,64,70]

- Linear Kernel: This is used for linearly separable data.
- Polynomial Kernel: Suitable for data that polynomial curves or surfaces can separate.
- Radial Basis Function (RBF) Kernel: Effective for complex, non-linear data.
- Sigmoid Kernel: Suitable for data with sigmoid-shaped decision boundaries.

Choosing the right kernel function is crucial for achieving optimal performance.

Step 6. Model Evaluation: After training the SVM model, it must assess its performance on a separate test dataset. The following are typical evaluation measures for categorization tasks [52,64,70]:

- Accuracy: The percentage of cases that were accurately categorized.
- Precision: It is measured as a ratio of real positives to all anticipated positives.
- Recall: The ratio of actual positive and true positive results.
- F1-Score: The harmonic mean of recall and precision strikes a balance between the two.
- ROC-AUC: The area beneath the Receiver Operating Characteristic curve gauges a model’s capacity for class distinction.

These metrics provide insight into the SVM model’s performance and determine whether it satisfies the requirements for occupancy detection.

Step 7. Prediction and Decision: Once trained, analyzed, and determined to meet performance criteria, the SVM model can predict occupancy on new, unseen data. Based on the learned decision boundary and the relevant attributes, the model will determine whether a space is occupied.

SVM for occupancy detection involves selecting relevant features, training the model to find a suitable decision boundary, handling non-linearity with kernel functions if needed, evaluating its performance, and finally using it for real-time predictions. The choice of features, kernel, and evaluation metrics should be carefully considered to build an effective occupancy detection system [52,64,70].

SVM complexity in occupancy detection involves choosing an appropriate kernel (e.g., linear, polynomial, radial basis function), optimizing hyperparameters like regularization (C) and kernel parameters, and addressing scalability issues for large datasets. Achieving a balance among these factors is crucial for enhancing the performance of SVM inaccurate occupancy predictions. Some advantages, disadvantages, and applications of the SVM algorithm in smart buildings are explained in Table 8 [52,64,70,71].

#### 5.2.2. K-Nearest Neighbour (KNN)

**Figure 7:**
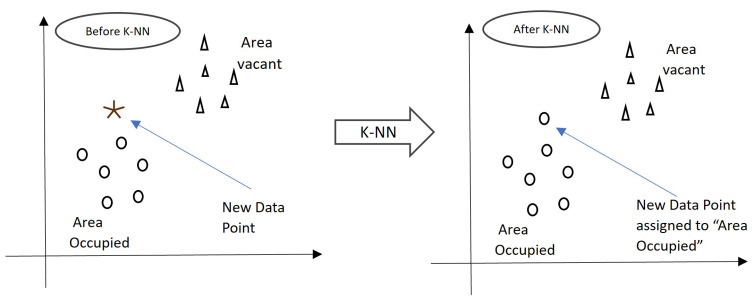
Occupancy classification using the KNN Algorithm [71].

The KNN machine learning technique is straightforward and intuitive and may be used for classification and regression applications [72,73]. In occupancy detection, KNN can be applied to determine whether a space (e.g., a room) is occupied based on its proximity to labeled data points in the feature space [72,73]. Figure 7 shows the occupancy classification using the KNN algorithm.

Next, we explain the mathematical explanation of the KNN method. Mathematical Explanation of KNN: Assume we have the following notations [10]: *X* is the set of feature vectors in the training dataset. *Y* is the corresponding vector of class labels (1 for “Occupied” and −1 for “Not Occupied”). $X_{\mathrm{new}}$ is the new feature vector that needs to be classified. *k* is the number of nearest neighbors to consider. *d*$(X_{1},X_{2})$ is the distance function between two feature vectors $X_{1}$ and $X_{2}$. *N* is the total number of data points in the training dataset.

Calculate distances: Determine the difference between the training dataset’s data points and the new feature vector, $X_{\mathrm{new}}$

(8) $$\begin{array}{c} \mathrm{Distance}\left(X_{\mathrm{new}},X_{i}\right)=d\left(X_{\mathrm{new}},X_{i}\right)\;\mathrm{for}\;i=1,2,\ldots ,N \end{array}$$

Find nearest neighbors: Choose the *k* data points that are closest to $X_{\mathrm{new}}$ in terms of distances: Let $X_{1},X_{2},\;\ldots ,X_{k}$ be the k data points with the smallest distances.

Majority voting: Among the k nearest neighbors, count the occurrences of each class label and classify $X_{\mathrm{new}}$ as the class label that has the highest count:

(9) $$\begin{array}{c} \sum_{i=1}^{k}Y\left(i\right)>\frac{k}{2}\;\mathrm{Classify}\;X_{\mathrm{new}}\;\mathrm{as}\;“\mathrm{Occupied}.” \end{array}$$

(10) $$\begin{array}{c} \sum_{i=1}^{k}Y\left(i\right)<=\frac{k}{2}\;\mathrm{Classify}\;X_{\mathrm{new}}\;\mathrm{as}\;“\mathrm{Unoccupied}.” \end{array}$$

where (Y(i)) is the *i*-th nearest neighbor’s class label.

The distance function $\left(d\left(X_{1},X_{2}\right)\right)$ can be Euclidean distance, Manhattan distance, or any other appropriate distance metric depending on the data and problem. For instance, Euclidean distance between two feature vectors $\left(X_{1}\right)$ and $\left(X_{2}\right)$ in a *n*-dimensional space is:

(11) $$\begin{array}{c} d\left(X_{1},X_{2}\right)=\sqrt{\sum_{j=1}^{n}{\left(X_{1j}-X_{2j}\right)}^{2}} \end{array}$$

where $X_{1j}$ and $X_{2j}$ are the *j*-th components of $X_{1}$ and $X_{2}$, respectively [10].

The (k) value influences the decision boundary and can impact the classifier’s performance. A larger (k) smooths the decision boundary, potentially leading to overgeneralization, while a smaller (k) might make the classifier sensitive to noise.

Step 3. Choosing the Value of K: The value of *K* is a critical hyperparameter in KNN. It determines how many nearest neighbors will be considered when making predictions. Here is a deeper look:

- Small *K*: A small value of *K* (e.g., K=1) can lead to a noisy decision boundary. The model may be overly sensitive to individual data points, resulting in erratic predictions and vulnerability to outliers.
- Large *K*: A large value of K (e.g., K=20) can lead to over-smoothing of the decision boundary. The model may ignore local patterns in the data and produce overly generalized predictions.

The features of the data and the current challenge will determine the appropriate value of *K*. Cross-validation and other experimental and validation methods are frequently used.

Step 4. Distance Metric:

- KNN uses a distance metric to assess how similar or dissimilar data points in the feature space are to one another [10,72,73]. The choice of distance metric may considerably impact the algorithm’s performance. Here are some typical distance metrics:
- Euclidean distance: This calculates the straight-line distances in Euclidean space between two places. For continuous, numerical properties, it is appropriate.
- Manhattan distance: It determines the total absolute differences along each dimension and is referred to as the L1 norm or city block distance. Features with varied scales or units are helpful.
- Cosine similarity: The cosine of the angle between two vectors is measured using this metric. It is frequently used for text data or where the direction of the vectors is more crucial than their size.

The distance metric should be selected based on the data’s characteristics and the problem’s scope. Experimentation may be required to figure out which metric performs the best.

Step 5. Model Training: In KNN, the training phase is not typical model training as seen in other algorithms. Instead, the algorithm simply memorizes the training dataset. It stores the feature vectors and their corresponding class labels so that it can use them during the prediction phase [10].

Step 6. Prediction: KNN determines the distance between the new point and every other point in the training dataset using the selected distance metric while making predictions for a new, unseen data point. This is how it goes:

- Determine the distance between the new data point and every point in the training dataset.
- Choose the K data points with the shortest distances between them (the closest neighbors).
- Count the frequency of each class among the K closest neighbors while doing classification tasks (like occupancy detection).
- Designate the projected class for the new data point as the one with the highest frequency.

Step 7. Model Evaluation: We must evaluate the KNN model’s performance after making predictions for the test dataset. Accuracy, precision, recall, F1-Score, and ROC-AUC, defined as before, are common classification measures [10,72,73].

KNN is a desirable choice for occupancy detection due to its simplicity and capacity for dealing with nonlinear decision limits. With large datasets, it can be computationally expensive, and the selection of K and the distance measure is essential for achieving the best results. Experimentation and hyperparameter tuning are often required to fine-tune the KNN model for the specific occupancy detection task [10,72,73].

KNN complexity in occupancy detection involves choosing a suitable distance metric, determining the optimal k value, and addressing storage challenges since KNN requires storing the entire dataset for reference during predictions. Balancing these factors is crucial for enhancing the accuracy of KNN in occupancy detection. Some advantages, disadvantages, and applications of the KNN algorithm in smart buildings are explained in Table 9 [10,71,72,73].

#### 5.2.3. Random Forest

**Figure 8:**
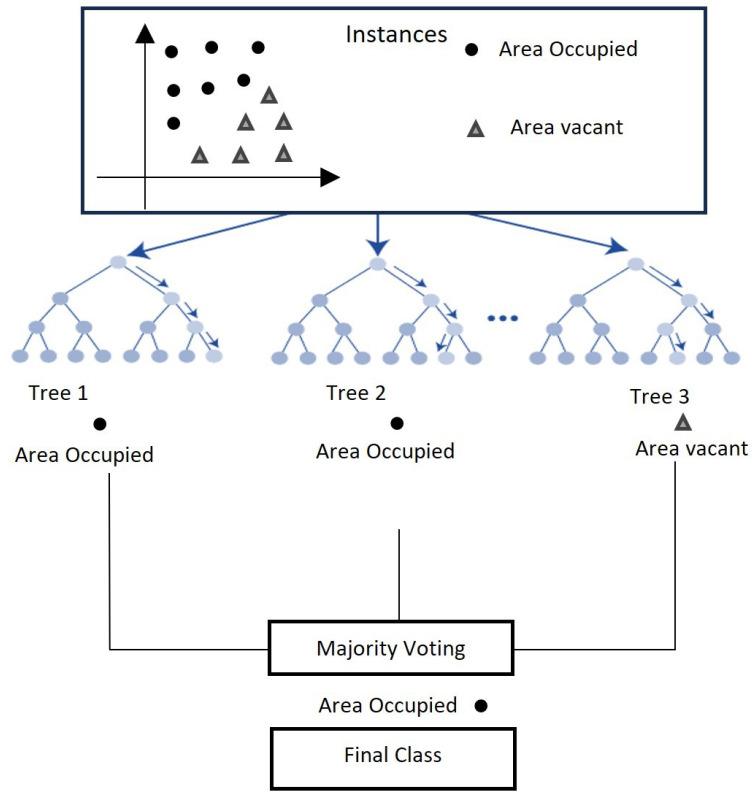
Occupancy classification using the RF algorithm [71].

An ensemble learning system called Random Forest (RF) integrates different decision trees to provide more accurate predictions [47,74]. In occupancy detection, Random Forest can determine whether a space (e.g., a room) is occupied based on various features and attributes. Figure 8 shows occupancy classification using the RF algorithm.

Mathematical Explanation of RF:

Assuming we have the notations as follows [47,74,75]:

- *N* is the number of samples.
- $X_{i}$ are the feature vectors like time of day, temperature, humidity, etc.
- $Y_{i}$ is the binary class label.
- *M* is the decision trees (hyperparameter), each trained independently on a different subset of the data.

Bootstrapping (Data Randomization):

For each decision tree in the method (from m=1 to):

- Create a bootstrapped sample $D_{m}$ by randomly selecting *N* samples from the original dataset with replacement.
- The size of $\left(D_{m}\right)$ is the same as the original dataset but may contain duplicates.

Feature Randomization:

- A choose a subset of the features $F_{m}$ at random to be considered for splitting at each decision tree node.
- Let K represent how many features were chosen at random. Sqrtp features, where *p* is the total number of features, is a popular option.
- The selection of $F_{m}$ is different for each tree, introducing feature diversity.

Decision Tree Construction:

For each tree *m* in the forest

- Build the decision tree iteratively by selecting the optimal feature Fm to divide the data into subsets according to a given criterion (such as Gini impurity or information gain).
- The feature $F_{m}$ is chosen at each node to either increase data acquisition or minimize impurity.
- A predetermined stopping criterion (such as a maximum depth or a minimum number of samples per leaf) is reached as the tree grows.

Voting for Classification:

- To predict the occupancy of a new data point $X_{\mathrm{new}}$ with features $X_{1},X_{2},\;\ldots ,X_{m}$, pass it through each of the *M* decision trees.
- Each tree m makes a prediction $Y_{m}$ (1 for “Occupied” or −1 for “Not Occupied”).
- The final prediction $Y_{\mathrm{new}}$ is defined by majority voting:

(12) $$\begin{array}{c} Y_{\mathrm{new}}=\mathrm{mode}\left(Y_{1},Y_{2},\ldots ,Y_{m}\right) \end{array}$$

Aggregation:

- The combined outcome of all the decision trees in the Random Forest is represented by the final prediction, $Y_{\mathrm{new}}$.
- A majority vote guarantees that the ensemble’s consensus will serve as the foundation for the final projection.

The calculations of impurity or information gain to determine the optimal feature to partition the data at each node of the decision trees is one of the fundamental mathematical ideas in Random Forests. Additionally, data and feature selection randomization ensure diversity among the trees, reducing overfitting and improving the model’s generalization performance for occupancy detection [47,74,75].

Step 3. Ensemble of Decision Trees: An ensemble of decision trees is built using the ensemble learning technique known as Random Forest during the training stage. Here is a deeper look at this step:

- Random Subsets: By training each decision tree on a random subset of the training data and a random selection of characteristics, Random Forest adds diversity to its ensemble. Through this procedure, overfitting is lessened, and the model is improved.
- Robust Predictions: Because Random Forest aggregates predictions from multiple trees, it is less prone to overfitting than individual decision trees. This ensemble approach improves generalization and the overall predictive performance.

Step 4. Bagging (Bootstrap Aggregating): The randomly selected subsets of data and features for each tree in Random Forest are produced via a process known as bagging (Bootstrap Aggregating). Here is how it works:

- Bootstrap Samples: Bagging involves repeatedly sampling the training data with replacement. This creates multiple bootstrap samples, each of which may contain duplicate instances and exclude some data points. Each bootstrap sample is used to train a separate decision tree.
- Random Feature Subset: Random Forest additionally chooses a random subset of characteristics for each tree in addition to randomizing the data. This further increases diversity by ensuring that several trees are exposed to various subsets of data and characteristics.

Step 5. Decision Tree Construction: The Random Forest ensemble’s decision trees are built using these principles [47,75]:

- Recursive Splitting: The data is recursively split into subsets according to the chosen features, and then the decision tree is constructed. This procedure continues until a stopping requirement, such as obtaining a minimum number of samples in a leaf node or reaching a maximum tree depth, is fulfilled.
- Impurity Minimization: The method seeks to maximize information gain or minimize impurity at each decision tree node. Entropy and Gini impurity are two popular impurity measurements. The splits are picked to aid in distinguishing between the classes (occupied or unoccupied) by increasing the purity of the resulting subsets.
- Leaf Nodes: The leaf nodes of the decision tree represent the final predictions for different combinations of feature values. For occupancy detection, these predictions will be whether a space is occupied or unoccupied.

Step 6. Prediction: Each decision tree in the Random Forest is used to classify a new, unobserved data point (identify its occupancy status). Here is how the prediction method functions [47,75]:

- Individual Tree Predictions: Each decision tree produces its prediction based on the new data point’s features.
- Aggregation: A majority vote (classification) or average (regression) of the individual tree predictions yields the final prediction for the new data point. The majority vote will determine whether the place is occupied or vacant in the context of occupancy detection.

Step 7. Model Evaluation: After making predictions for the test dataset, the Random Forest model’s performance is evaluated using standard classification metrics. These metrics include accuracy, precision, recall, F1-Score, and ROC-AUC defined as before.

The strengths of Random Forest include handling complex data, capturing nonlinear correlations, and using an ensemble technique to reduce overfitting [47,74,75]. It is a versatile algorithm well-suited for occupancy detection when we have diverse and noisy data. Proper hyperparameter tuning and feature selection can improve performance for this specific task.

Random Forest complexity in occupancy detection involves controlling tree depth to prevent overfitting, balancing the number of trees for accuracy and computational efficiency, and understanding feature importance for effective model interpretation. Balancing these aspects is key to optimizing Random Forests’ performance in accurate occupancy predictions. Some advantages, disadvantages, and applications of the RF algorithm in smart buildings are explained in Table 10 [47,71,74,75].

#### 5.2.4. Deep Learning

Machine learning’s area of Deep Learning focuses on using Artificial Neural Network (ANN) and Convolutional Neural Network (CNN), etc., to solve complex problems [65,76]. In occupancy detection, Deep Learning algorithms, particularly neural networks, can be employed to determine whether a space (e.g., a room) is occupied or not based on various features and attributes [68,77].

Here is how occupancy detection using Deep Learning methods works:

Step 3. Neural Network Architecture: Deep Learning models frequently have many layers of coupled neurons. The feature data are entered into the first layer, the input layer. The data are subjected to numerous mathematical procedures in the hidden layers that come after. The output layer is the last, and it generates the prediction (occupied or unoccupied) based on the data that has been processed [65,78].

Step 4. Activation Functions: Every neuron in a neural network has an activation function, which gives the model non-linearity. Activation functions often utilized include tanh, sigmoid, and the rectified linear unit (ReLU). Which activation functions are used depends on the network architecture and the data type [65,78].

Step 5. Model Training: The neural network learns from the training data during training by modifying its weights and biases through an optimization procedure. Backpropagation and gradient descent are commonly used for this, and the model works to minimize a loss function that measures the discrepancy between the anticipated outputs and the true labels [65,78].

Step 6. Overfitting Prevention: Deep Learning models are particularly prone to overfitting when working with expansive and complicated networks. To avoid overfitting and enhance generalization, dropout, regularization, and early halting are often used [65,78].

Step 7. Model Evaluation: A separate test dataset is used to evaluate the Deep Learning model after training in order to assess its performance using common classification metrics, including accuracy, precision, recall, F1-score, and ROC-AUC [65,78].

Step 8. Prediction: By running the features through the network and collecting the output from the final layer, the Deep Learning model may be used to predict the occupancy status of new, unobserved data once it has been trained and validated [65,78].

Deep Learning algorithms, particularly deep neural networks, effectively identify intricate patterns and connections in data [68,77]. They are ideally suited for high-dimensional data applications, such as occupancy detection. Since they can automatically develop hierarchical representations from the input, however, Deep Learning models frequently need a lot of data and computer resources for training. Because of their complexity, interpretability can be difficult [68,77]. Nevertheless, Deep Learning methods can be a good option for occupancy identification when the data are plentiful, and the issue is complex.

Some common methods across most Deep Learning algorithms, including the four major methods we mentioned, are Feedforward Neural Network (FNN), Convolutional Neural Network (CNN), Recurrent Neural Network (RNN), and Long Short-Term Memory (LSTM)).

Feedforward Neural Network

**Figure 9:**
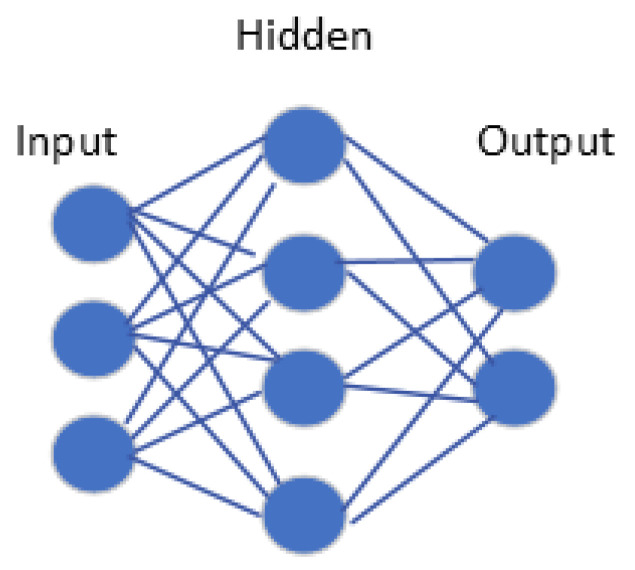
FNN architecture with 3 layers [80].

Artificial neural networks are built on a foundation of FNNs, also called multilayer perceptrons. A hidden layer or layers, an output layer, and an input layer make them up. Without feedback loops, information moves from input to output in a single direction [65,66,79]. Each connection between neurons in one layer and neurons in the layer above it carries a certain weight. FNNs are a key machine learning component because they work effectively for various tasks, such as classification, regression, and pattern recognition [66,79]. Figure 9 shows the FNN architecture with three layers.

In order to understand FNN better, next, we introduce its mathematical explanation.

Data Representation:

- Let *X* be the input feature vector representing various features (e.g., time of day, temperature, humidity) for occupancy detection.
- *y* is the corresponding binary class label: y=1 for “Occupied” and y=0 for “Not Occupied.”

A mathematical representation of a feedforward neural network is a series of transformations from the input to the output layer.

- Let *L* represent the total number of layers, where L=3 in a simple network (input layer, hidden layer, output layer).
- The output of each layer can be mathematicallyrepresented as:
  (13) $$\begin{array}{c} z^{\left(l\right)}=W\left(l\right)\times a\left(l-1\right)+b\left(l\right) \end{array}$$
  (14) $$\begin{array}{c} a^{\left(l\right)}=\sigma \left(z^{\left(l\right)}\right) \end{array}$$
  where
  – *l* represents the layer index.
  – $z^{\left(l\right)}$ is the weighted sum of inputs plus the bias for layer *l*.
  – a(l) is the output of layer *l* after applying the activation function $\sigma (z^{\left(l\right)})$.
  – W(l) is the weight matrix for layer *l*, and b(l) is the bias vector for layer *l*.
  – $\sigma (z^{\left(l\right)})$ is typically a non-linear activation function like the sigmoid or ReLU.

Feedforward Process:

- The feedforward process calculates the predicted output *Y* based on the input *X* and the learned weights and biases.
- *l* represents the output layer, and Ẏ is the predicted probability of occupancy (between 0 and 1).

Loss Function:

- The binary cross-entropy loss, often known as the loss function for binary classification, is defined as:

(15) $$\begin{array}{c} \mathrm{Loss}(y,Y)=-[y\log(Y)+(1-y)\log(1-Y)] \end{array}$$

- *y* is the true class label (0 or 1), and *Y* is the predicted probability of occupancy.

Training—Backpropagation:

- It is possible to minimize the average loss across the entire training dataset during training,
- By computing the gradients of the loss concerning the network’s parameters (weights and biases) and updating these parameters using an optimization algorithm like gradient descent [66,81].
- The gradient of the loss relative to the activation of the output layer $a^{\left(L\right)}$ is:

(16) $$\begin{array}{c} \frac{\partial \mathrm{Loss}}{\partial a^{\left(L\right)}}=\frac{Y-y}{Y(1-Y)} \end{array}$$

- Backpropagation involves propagating this gradient backward through the layers, computing gradients for each layer, and using them to update the weights and biases [66,81].

Regularization Techniques: In order to avoid overfitting, which happens when the model fits the training data too closely and performs badly on unknown data, regularization is an important machine learning approach, including FNN. L1 and L2 regularization are two widely used regularization methods:

L1 Regularization (Lasso): A penalty term corresponding to the absolute values of the weights is added to the loss function via L1 regularization. It chooses a subset of the most crucial characteristics by encouraging the model to have sparse weight values. Removing less important features can help decrease the danger of overfitting in occupancy detection [66,79,81].

L2 Regularization (Ridge): A penalty term proportional to the square of the weights is added to the loss function using L2 regularization. It promotes a smoother, more generalized model, which deters the model from having excessively big weights and aids in preventing overfitting.

In occupancy detection, regularization techniques can be used on the FNN to manage the model’s complexity and enhance its capacity to generalize to new data.

Batch Processing: In FNN training, batch processing is a common practice. Instead of updating the model’s weights after processing each data point (as in online learning) or after processing the entire dataset (as in batch learning), training is performed on mini-batches of data [66,79,81]. Here is how it works:

Mini-Batches: Mini-batches, which are smaller subsets of the training dataset, are created. A hyperparameter that can be modified is the mini-batch size (also known as batch size) [66,81].

Batch Dimension: Introducing mini-batches adds a new dimension (the batch dimension) to the calculations in FNN training. This means that the forward and backward passes (calculating predictions and gradients) are simultaneously done for multiple data points.

Gradients for the Entire Batch: For each data point in the mini-batch, gradients of the loss concerning the model parameters (weights and biases) are calculated during the backward pass. This aids in weight updates that are more reliable and effective.

In occupancy detection, batch processing can speed up the training process and lead to a more stable model convergence. It can also allow for more efficient GPU utilization when training deep neural networks.

Optimization:

The method of optimization involves repeatedly changing the model’s weights and biases in order to reduce the loss function. There are several optimization algorithms accessible, and each has benefits and drawbacks. Common optimization algorithms used in FNN training include:

Stochastic Gradient Descent (SGD): Each iteration of SGD involves updating the model’s weights depending on the gradient of the loss function for a single randomly selected data point. It introduces stochasticity into the optimization process, which can help escape local minima [81].

Adam (Adaptive Moment Estimation): Adam is a learning rate optimization algorithm that adapts to changing gradients by changing the learning rate for each parameter. It combines the benefits of momentum-based approaches and SGD [66,81].

Prediction:

After training, the FNN can make predictions on new data points. The process involves applying the learned weights and biases to the input features and computing the final output. In occupancy detection, the FNN can take in environmental features (e.g., temperature, humidity, time of day) and predict whether a room is occupied based on the learned patterns from the training data [65,66,81].

Regularization, batch processing, optimization, and prediction are essential components of FNNs for occupancy detection. These techniques help create accurate models on the training data and generalize well to unseen data, which is crucial for real-world applications [66,81]. The choice of specific techniques and hyperparameters should be based on the characteristics of the dataset and the objectives of the occupancy detection task.

FNN complexity in occupancy detection involves designing architecture parameters, including hidden layers and activation functions, optimizing training through backpropagation and weight updates, and ensuring generalization by mitigating overfitting with regularization techniques. Balancing these factors is key to optimizing the performance of neural networks in accurate occupancy predictions. There are some advantages, disadvantages, and applications of the FNN algorithm in smart buildings explained in Table 11 [65,66,79,80,81].

#### Convolutional Neural Network

**Figure 10:**
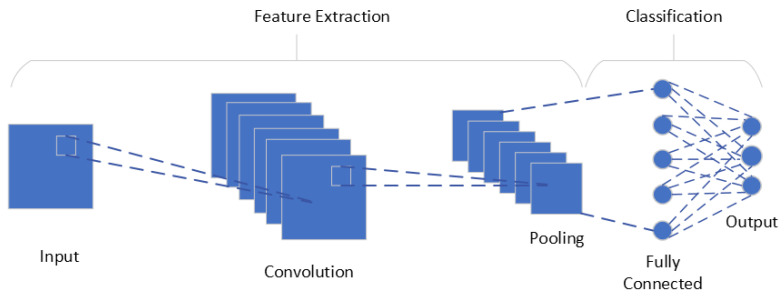
CNN architecture with five layers [82].

CNNs are specialized neural networks made for processing data that can be organized into grids, like photographs and spatial information. When conducting tasks like picture identification, they use convolutional layers to find local patterns in the input [65,77]. CNNs excel in automatically learning hierarchical characteristics, starting with basic edges and textures and working their way up to complicated object sections. Downsampling is accomplished via pooling layers; fully linked layers make final predictions. CNNs have been revolutionary in computer vision but can also be adapted for sequential data, like time series. Figure 10 shows the CNN architecture with five layers.

In order to understand CNN better, next, we introduce its mathematical explanation.

Mathematical Explanation:

- CNNs are generally made for analyzing data with a grid structure, such images. However, they can also be adapted to handle sequential or time-series data, which is common in occupancy detection applications [65,77].

Input Layer:

- In occupancy detection, input data often include sequential features, such as time, temperature, humidity, and other environmental variables.
- These sequential features can be represented as a 1D time series: $X=[x_{1},x_{2},\ldots x_{n}]$, where *n* is the number of time steps or data points [77].

Convolutional Layer:

- The core component of a CNN is the convolutional layer. In occupancy detection, a 1D convolutional layer can be applied to capture local patterns in the sequential data.
- The convolution operation extracts features by applying a set of learnable filters to the input data.
- For each filter, the convolution operation computes a weighted sum of the input values within a window of size *k* (where *k* is the filter size):

(17) $$\begin{array}{c} z_{j}^{\left(1\right)}=\sum_{i=1}^{k}w_{i}^{\left(1\right)}x_{j+i-1}+b_{j}^{\left(1\right)} \end{array}$$

- After the weighted sum, an activation function.

(18) $$\begin{array}{c} a_{j}^{\left(1\right)}=\sigma \left(z_{j}^{\left(1\right)}\right) \end{array}$$

- This process is repeated for each filter, creating a feature map.

Pooling Layer:

- After convolution, pooling layers are often used to downsample and retain essential features while reducing the spatial dimensionality of the data [65,77].
- Max-pooling is a popular pooling approach where the highest value found in a particular local region is kept while the others are discarded.

Fully Connected Layer:

- The next layer in the network after the convolutional and pooling layers is usually one or more fully linked layers. The output of the previous layers is flattened into a vector and fed into these fully connected layers [65,77].
- The hidden and output layers in a Feedforward Neural Network (FNN) are comparable to these layers.

Training:

- Gradient descent with backpropagation is used to optimize the weights and biases of the network during the training of a CNN for occupancy detection.
- Depending on the specific occupancy detection task, the loss function is selected (for example, binary cross-entropy loss for binary classification).
- The CNN gains the ability to identify and predict relevant patterns and features from the input time series data during training.

A CNN can be advantageous in occupancy detection when the environmental data’s temporal dependencies and local patterns are crucial for accurate prediction [77]. The 1D convolutional layers are capable of capturing these patterns efficiently. However, the architecture and hyperparameters of the CNN should be carefully tuned to match the characteristics of the occupancy dataset for optimal performance [65,77].

CNN complexity in occupancy detection involves balancing layer depth for feature complexity with computational resources, selecting appropriate kernel sizes and strides for effective feature extraction, and deciding on pooling methods like max-pooling or average-pooling. Achieving this balance is crucial for optimizing CNN performance in accurate occupancy predictions. The advantages, disadvantages, and applications of the CNN algorithm in smart buildings are explained in Table 12 [65,77,82].

#### Recurrent Neural Network

**Figure 11:**
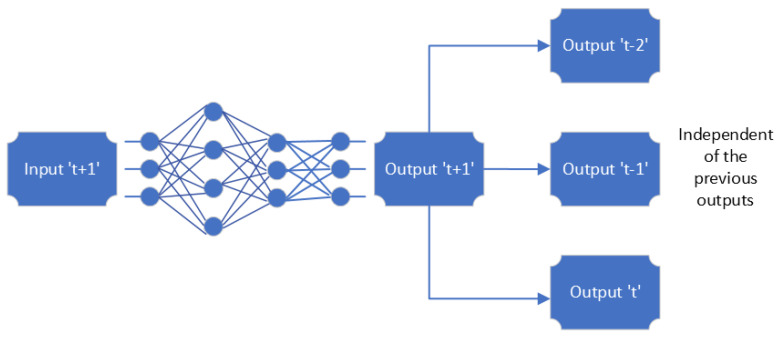
RNN Architecture [85].

RNNs are designed specifically to handle sequential data, where the timing and order of the information are crucial. Recurrent connections, not present in FNNs or CNNs, enable information to pass from input to output and through loops that feed data from earlier time steps into the current step [65,83]. This qualifies them for jobs like time series analysis, speech recognition, and natural language processing. RNNs can recognize temporal connections and context in the data since they have a memory of past inputs [83]. However, due to problems with disappearing gradients in lengthy sequences, advanced variations, such as Long Short-Term Memory (LSTM) [84] and Gated Recurrent Unit (GRU) networks, have been developed. Figure 11 shows the RNN architecture with seven layers.

Next, we introduce the mathematical explanation of the RNN method. Mathematical explanation for RNNs in occupancy detection:

Input Layer:

- In occupancy detection, the input layer receives sequential data representing features at different time steps [65,83].
- Input features: $\left[X=x_{1},x_{2},\;\ldots ,\;x_{n}\right]$ where *n* is the number of time steps.
- Each $x_{1}$ represents a feature at a specific time step *i*.
- These features could include environmental variables like temperature, humidity, light intensity, and time of day, which can be relevant for occupancy detection.

Recurrent Layer:

- The recurrent layer in RNNs is designed to capture temporal dependencies in sequential data.
- It keeps hidden states constant over time, enabling the network to recall data from earlier time steps and apply it to predictions at the present step [65,83].
- The hidden state at each time step (*h*(*t*)) is computed as follows:
  (19) $$\begin{array}{c} h\left(t\right)=\sigma (W_{hh}h\left(t-1\right)+W_{xh}x\left(t\right)+b_{h}) \end{array}$$
  where:
  – h(t) is the hidden state at time step *t*.
  – σ is a non-linear activation function (e.g., sigmoid or hyperbolic tangent).
  – $W_{hh}$ and $W_{xh}$ are weight matrices that control the flow of information from the previous hidden state $h_{\left(t-1\right)}$ and the current input x(t) to the current hidden state.
  – $b_{h}$ is the bias term.

- By updating the hidden state at each time step depending on both the current input and the knowledge from the past, this recurrent connection enables the network to represent data sequences.

Output Layer:

- The output layer of an RNN is in charge of making predictions, much like the output layer in a Feedforward Neural Network (FNN) [65,83].
- The specific occupancy detection task determines the output layer’s architecture. Typically, a single output neuron with a sigmoid activation function is utilized for binary occupancy detection (occupied or not).

Training:

- Training an RNN for occupancy detection involves optimizing the weights $W_{hh}$ and $W_{xh}$ and biases $b_{h}$ using backpropagation through time (BPTT) and gradient descent [65,83].
- The loss function is chosen based on the task. For binary occupancy detection, binary cross-entropy loss is often used.

Evaluation: To compare the performance of the RNN model for occupancy detection, we would typically:

- Train the model on a training dataset to compare the performance of the RNN model for occupancy detection.
- Validate the model using a validation dataset (and, if necessary, adjust the hyperparameters).
- Test the model on a test dataset to determine how well it performs.
- To measure how well the model is predicting occupancy status, use metrics like accuracy, precision, recall, F1-score, and ROC-AUC.

RNNs are well-suited for occupancy detection tasks that involve sequential data, as they can capture temporal dependencies in the input features. The hidden state and recurrent connections allow RNNs to model and make predictions based on sequences, making them a valuable tool for tasks where the order and timing of events matter, such as occupancy detection in smart buildings [65,83].

RNN complexity in occupancy detection includes capturing temporal dependencies for long-range modeling, addressing vanishing gradient issues during training, and handling variable-length sequences. Balancing these factors is essential for optimizing RNN performance in accurate occupancy predictions. Some advantages, disadvantages, and applications of the RNN algorithm in smart buildings are explained in Table 13 [65,80,83,85].

#### Long Short-Term Memory

Long Short-Term Memory (LSTM) is a form of recurrent neural network (RNN) architecture created to successfully capture long-range dependencies in sequential data while overcoming the drawbacks of conventional RNNs, such as the vanishing gradient problem [65,66,76,77,86,87]. Natural language processing, speech recognition, time series analysis, and occupancy detection are just a few of the numerous uses for LSTMs.

The capacity of LSTMs to retain and retrieve data over long sequences is one of their important characteristics. They accomplish this by establishing an internal structure that is more complicated than typical RNNs, including using gates to regulate the movement of information inside the network. These gates control the flow of information into and out of memory cells by activating sigmoid and tanh (hyperbolic tangent) functions [77]. Input gate, forget gate, output gate, memory cell state, and hidden state are all components of the LSTM architecture.

**Figure 12:**
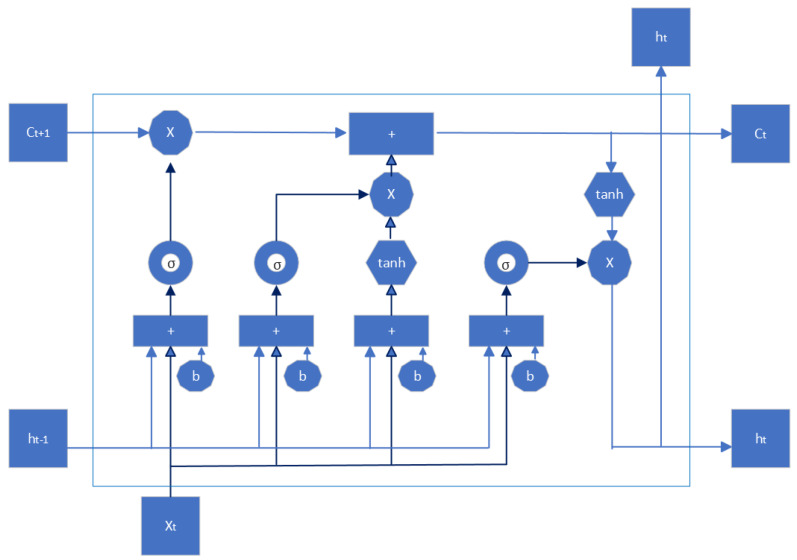
LSTM architecture [88].

Because LSTMs can identify and retain intricate patterns and connections in data sequences, LSTMs have become a potent tool for modeling sequential data [65,77]. LSTMs are useful for building management, energy efficiency, and smart building systems because they can be used in occupancy detection to analyze and predict occupancy status based on past data. The RNN method’s mathematical justification is then shown. Figure 12 shows the LSTM architecture.

Mathematical explanation of LSTM:

Input Layer:

In occupancy detection, the input layer receives sequential data, where each element in the sequence corresponds to a time step. These sequences could represent time series data of various environmental variables (e.g., temperature, humidity, light intensity) and other relevant features. The LSTM network processes these sequential inputs, allowing it to learn and capture patterns over time [77].

We can represent the input sequence as: $X=[x_{1},x_{2},\;\ldots ,\;x_{t}]$ where *t* is the number of time steps.

LSTM Layer: The LSTM layer consists of LSTM cells, each with the following mathematical operations:

- Input Gate $i_{t}$: identifies the amount of new data added to the cell state $c_{t}$.

(20) $$\begin{array}{c} i_{t}=\sigma \left(W_{xi}x_{t}+W_{hi}h_{t-1}+W_{ci}c_{t-1}+b_{i}\right) \end{array}$$

- Forget Gate $f_{t}$: establishes which data from the previous cell state $c_{\left(t-1\right)}$ should be forgotten.

(21) $$\begin{array}{c} f_{t}=\sigma \left(W_{xf}x_{t}+W_{hf}h_{t-1}+W_{cf}c_{t-1}+b_{f}\right) \end{array}$$

- Output Gate $o_{t}$: controls how much information is revealed to the output and how the cell status affects the hidden stat $h_{t}$.

(22) $$\begin{array}{c} o_{t}=\sigma \left(W_{xo}x_{t}+W_{ho}h_{t-1}+W_{co}c_{t-1}+b_{o}\right) \end{array}$$

- Cell State $c_{t}$: based on the input gate $i_{t}$ and forget gate $f_{t}$, it updates the memory of the cell.

(23) $$\begin{array}{c} c_{t}=f_{t}\cdot c_{t-1}+i_{t}\cdot \tanh\left(W_{xc}x_{t}+W_{hc}h_{t-1}+b_{c}\right) \end{array}$$

- Hidden State $h_{t}$: This is the output of the LSTM cell’s output, influenced by the cell state $c_{t}$ and the output gate $o_{t}$.

(24) $$\begin{array}{c} h_{t}=o_{t}\cdot \tanh\left(c_{t}\right) \end{array}$$

Output Layer: After processing through the LSTM layer, the network can have additional layers and output neurons tailored to the occupancy detection task. Typically, a single output neuron with a sigmoid activation function is utilized for binary occupancy detection (occupied or not) [77].

Training:

- LSTM training involves optimizing weights and biases using backpropagation through time (BPTT) and gradient descent to minimize a suitable loss function.
- The loss function to use (such as binary cross-entropy loss) depends on the unique occupancy detection problem.
- LSTMs are particularly effective in capturing and modeling temporal dependencies, making them well-suited for occupancy detection, where occupancy status often depends on historical data patterns [65,77].

Due to LSTM’s ability to recognize and retain intricate patterns and correlations in data sequences, LSTMs have emerged as a potent tool for modeling sequential data. For applications in building management, energy efficiency, and smart building systems, LSTMs are useful in occupancy detection because they can be used to analyze and predict occupancy status based on past data [65,76,77].

LSTM network complexity in occupancy detection involves managing cell state updates for information preservation, understanding the roles of gates (Forget, Input, Output) in controlling information flow and optimizing hyperparameters such as hidden units and layers. Achieving a balance in these aspects is crucial for optimizing the performance of the LSTM network’s inaccurate occupancy predictions. Some advantages, disadvantages, and applications of the RF algorithm in smart buildings are explained in Table 14 [65,66,76,77,86,87,88].

### 5.3. Comparison of Algorithms

The benefits and drawbacks of occupancy detection systems and algorithms are discussed in this section. Given the application context and accuracy requirements, we provide a comparative approach to aid researchers in selecting sensors and algorithms that are more suitable to implement. In order to provide a framework for evaluating occupancy detection systems, it is critical to consider several features, including sensor types, data processing techniques, occupancy resolution, and performance measurements.

#### 5.3.1. Comparison of Traditional Occupancy Detection Algorithms

This section presents a comprehensive comparison of the various occupancy detection algorithms identified within this study’s scope.

Table 15 compares the traditional occupancy detection algorithms identified within this study’s scope. Four distinct algorithms are under consideration, each harnessing different sensor types and exhibiting varying degrees of accuracy. These algorithms utilize PIR sensors, environmental sensors, smart meters, and sensor fusion in distinct ways, shaping their performance characteristics.

Firstly, we delve into the BOD algorithm, which relies on PIR sensors prominently while displaying a modest dependence on environmental sensors. Notably, the BOD algorithm achieves a commendably high level of accuracy in its occupancy detection tasks. Moving on to the FLOD algorithm, we observe a somewhat contrasting sensor utilization pattern. The FLOD algorithm emphasizes PIR sensors less while displaying a moderate reliance on environmental sensors. In line with its counterpart, BOD, FLOD also boasts high accuracy in its occupancy detection endeavors.

Next, we scrutinize the MOD algorithm, which exhibits a distinctive sensor utilization profile. MOD places a lower emphasis on PIR sensors, instead relying more heavily on environmental sensors. However, it should be noted that MOD’s accuracy level falls within the medium range, setting it apart from the aforementioned high-accuracy algorithms. Lastly, we examine the WOD algorithm, which presents yet another facet of sensor utilization. WOD displays a moderate reliance on PIR sensors, with a diminished reliance on environmental sensors, resulting in a comparatively lower accuracy level.

In the context of smart meter integration, it is noteworthy that both MOD and WOD algorithms incorporate smart meters, enhancing their functionality. Conversely, the BOD and FLOD algorithms do not incorporate smart meters into their occupancy detection methodologies. Furthermore, the MOD and FLOD algorithms use sensor fusion techniques, which enhance their performance by combining data from multiple sensor sources. In contrast, the BOD and WOD algorithms do not employ sensor fusion as part of their occupancy detection strategies.

This comparative analysis sheds light on the distinctive characteristics and sensor dependencies of the four occupancy detection algorithms, offering valuable insights into their strengths and weaknesses for different application scenarios.

#### 5.3.2. Comparison of Machine Learning Occupancy Detection Algorithms

Table 16 and Table 17 present a comparison of different algorithms based on the smart meter measurements [78] data used for occupancy detection within the smart building.

We need to assess these algorithms using the proper measures, such as accuracy, precision, recall, F1-score, and ROC-AUC, in order to compare the performances of SVM, RF, and KNN on occupancy data. SVMs are unique in machine learning, according to Table 16 because of their outstanding qualities. They deliver not only high accuracy but also exhibit impressive robustness. Additionally, SVMs boast a remarkable capability for rapid training while maintaining a commendably low demand for computational scalability.

KNN presents a more balanced tradeoff. It offers moderate accuracy, making it suitable for various scenarios, albeit with lower robustness. KNN’s memory requirements are relatively high, but its training time is minimal, rendering it a viable choice for certain applications.

Meanwhile, Random Forests shine with their high accuracy and robustness, making them a preferred choice for tasks where precision and resilience are paramount. While their training time is moderate, they have a noteworthy memory requirement, which should be considered in resource-constrained environments. Lastly, Deep Learning, the neural network-based approach, is hailed for its exceptional accuracy and robustness. However, these remarkable attributes come at a cost, as Deep Learning models typically demand substantial training time and memory resources.

The Comparison in Table 16 highlights that while Deep Learning and Random Forests offer top-tier accuracy and robustness, they require higher training time and memory resources investments. In contrast, SVM provide a commendable balance of high accuracy and robustness, coupled with more efficient memory and training requirements. KNN, with its moderate accuracy and resource-intensive memory demand, serves as a middle ground for applications where such characteristics align with the project’s goals.

As shown in Table 17, The choice of the best algorithm depends on specific goals and requirements for occupancy detection:

Accuracy-Focused: If the primary concern is overall accuracy, Random Forest performs best with the highest accuracy score (0.92).

Balanced Precision and Recall: If maintaining a balance between precision and recall is crucial, both Random Forest and KNN perform well, with similar precision and recall scores.

F1-Score Oriented: If we aim for a good F1-score, Random Forest is a strong choice as it has a high F1-score (0.92). Interpretability: If interpretability is important, KNN and SVM may be more straightforward, as decision boundaries are easier to understand.

Robustness: Consider the robustness of the model to noise and outliers. Random Forest is known for its robustness in handling noisy data.

As shown in Table 18, Comparing the performances of different neural network architectures (FNN, CNN, RNN, LSTM) on occupancy data involves evaluating various metrics, such as accuracy, precision, recall, F1-score, and ROC-AUC, to determine which model performs better for specific dataset and problem. The choice of the best model depends on factors like the nature of the data, the complexity of patterns, and the temporal dependencies involved in occupancy detection.

The LSTM model performs the best across all metrics, showing higher accuracy, precision, recall, F1-score, and ROC-AUC than other architectures. Here is why LSTM might excel:

Temporal Dependencies: Occupancy data often have strong temporal dependencies. LSTMs are explicitly designed to capture such dependencies, making them well-suited for this task.

Long-Range Patterns: If occupancy patterns depend on events occurring over extended periods, LSTMs can better capture these long-range patterns compared to simpler architectures like FNNs.

Sequential Information: LSTMs maintain memory over time, allowing them to leverage sequential information effectively. For instance, they can recognize patterns like “occupancy tends to increase during lunch hours”.

However, it is crucial to remember that the best model selection depends on the particular dataset and issue. Sometimes, a simpler model like an FNN may perform adequately, especially if the occupancy patterns are relatively straightforward. Experimentation and cross-validation are crucial to determining the most suitable model for the particular occupancy detection task.

## 6. Discussion

1. What are the incidents/scenarios where sensors can be confused or collect wrong information in smart buildings?

In the intricate landscape of smart buildings, deploying sensors for occupancy detection can encounter various incidents and scenarios that lead to confusion or the collection of erroneous information. One of the prevalent challenges arises from environmental factors and interference. Sensors, particularly light sensors, might misinterpret abrupt changes in natural light due to weather fluctuations as occupancy indicators, resulting in false readings. Similarly, variations in temperature or electromagnetic disturbances can confuse sensors, impacting their accuracy.

Another critical factor influencing sensor reliability is their placement and coverage within the building. Incorrect positioning or insufficient coverage can create blind spots, leaving certain areas unmonitored or inaccurately assessed for occupancy. Shadows cast by moving objects or temporary obstructions, like passing vehicles or swaying trees outside windows, can trigger motion sensors, leading to false occupancy alerts.

Human behavior adds another layer of complexity. Sensors may misinterpret non-traditional movements or activities, leading to discrepancies in occupancy data. Uncommon behaviors or irregular movement patterns might not align with the typical data these sensors are calibrated for, causing misinterpretations.

Moreover, technology limitations inherent to different sensor types can contribute to inaccuracies. For instance, infrared sensors might struggle to distinguish between occupancy and ambient temperatures matching body heat. Ultrasonic sensors could misinterpret echoes or bouncing signals, leading to flawed occupancy readings.

Furthermore, errors in data processing algorithms or inadequate calibration of sensor networks can result in false positives or negatives in occupancy detection. User interaction and manual overrides within the building systems might confuse sensors, causing discrepancies between actual occupancy status and the data collected.

Addressing these challenges requires a multifaceted approach involving meticulous sensor placement, employing diverse sensor types for cross-validation, routine maintenance, and calibration of sensors. Utilizing sophisticated data processing algorithms and ensuring user education to minimize disruptions that affect sensor accuracy are vital steps in mitigating these challenges within smart building environments. Regular system checks and updates play a pivotal role in maintaining the accuracy and reliability of occupancy detection systems.

2. Why do we need a complex algorithm like a machine learning algorithm for occupancy detection in smart buildings? Why is the simple solution or algorithm not working?

Occupancy detection in smart buildings might seem like a task that could be addressed with simple solutions or algorithms. However, the complexity of human behavior and environmental variability often demands more sophisticated approaches, such as machine learning algorithms.

Simple algorithms, such as threshold-based or rule-based systems, might struggle to account for the nuances of occupancy patterns. They often rely on fixed thresholds or predefined rules to determine occupancy status based on sensor readings. However, these simplistic approaches can falter in accommodating the variability of human behavior and diverse activities within a space. They might generate false positives or negatives, leading to inaccuracies in determining occupancy.

Machine learning algorithms, on the other hand, offer a more adaptable and dynamic approach. They can analyze vast amounts of sensor data and learn patterns and correlations that might not be apparent to rule-based systems. These algorithms can adapt to changing conditions and adjust their models based on real-time data, enhancing occupancy detection accuracy. They handle complex and non-linear relationships between various data points, allowing for a more nuanced understanding of occupancy patterns.

Moreover, machine learning algorithms can factor in multiple sensor inputs, considering a broader spectrum of data beyond simple binary readings. This multifaceted analysis enables them to differentiate between actual occupancies and environmental factors, such as temporary obstructions or varying light conditions, which might confuse simpler algorithms.

The use of machine learning also facilitates continuous improvement. These algorithms can continuously learn from new data, refining their models to improve accuracy and adapt to evolving occupancy patterns within smart buildings. They offer flexibility and adaptability that simpler algorithms might lack, making them better suited to handle the intricacies and variability inherent in occupancy detection in dynamic environments like smart buildings.

3. Do machine algorithms count the precise number of people in a smart building or focus on overall results? What specific data aid in the decision-making process during occupancy detection?

Machine learning algorithms analyze many sensor data to infer the overall occupancy status rather than focusing on exact headcounts. These algorithms process various data inputs collected from sensors throughout the building environment.

1. Motion Sensors: These sensors detect movement within designated areas. When triggered, they indicate activity, signaling the potential presence of occupants. However, they do not provide specific counts of individuals.

2. Light Sensors: Monitoring changes in light levels helps infer occupancy changes. Variations in lighting, especially in spaces adjusted based on occupancy, can indicate whether a room is likely occupied or unoccupied.

3. Temperature and Humidity Sensors: Though not directly linked to occupancy, these sensors can indirectly suggest changes due to human presence. For instance, increased body heat affecting room temperature or shifts in humidity levels might coincide with occupancy changes.

4. Sound or Wi-Fi Signals: In more advanced systems, sound sensors might detect noise associated with occupancy, while analyzing Wi-Fi signals could indicate the presence of devices carried by individuals [89].

Machine learning algorithms collectively use these diverse data inputs to understand occupancy patterns and trends. Instead of focusing on precise headcounts, these algorithms aim to identify correlations and patterns associated with occupancy across various sensor data.

The decision-making process involves inferring the likelihood of occupancy based on these identified patterns and data correlations. Algorithms continuously learn from historical data, adapting and improving their models to better predict occupancy states—occupied, unoccupied, or transitioning between states—within the building environment.

Ultimately, the goal is to decide the overall occupancy status of different areas or zones within the building. These algorithms enable smart building systems to automate actions such as adjusting lighting, HVAC systems, or security measures based on the inferred occupancy status. This leads to more efficient and responsive building management without focusing on individual headcounts.

## 7. Challenges and Future Works

The current occupancy detection methods discussed in this literature review are effective and efficient and have shown great potential in various applications such as building automation systems and security. However, to fully realize their potential, several issues raised by their deployment in the real world must be resolved. In this section, we will talk about several challenges and future directions for future studies that could be investigated to boost the precision, effectiveness, and affordability of occupancy detection in smart buildings.

First, one major challenge in deploying occupancy detection sensors and algorithms is the lack of standardization in the industry. Different sensors and algorithms may produce different results, making comparing and optimizing their performance difficult. Therefore, one future work is to build benchmarks for comping these methods, including dataset and evaluation benchmarks (including developing standardized testing procedures and protocols to evaluate the performance of occupancy detection sensors and algorithms).

Second, another challenge is the privacy concerns associated with all the sensors that may capture and store occupants’ data. The data collected by occupancy detection sensors may also be sensitive and require secure storage and processing. In the meantime, many sensors are built to be inexpensive to provide a wild deployment of occupancy detection to save power. Therefore, the adopted sensors may have limited computation and communication power. As one of the future works, it is crucial to ensure both data privacy and security using resource-limited sensors, and therefore, better algorithms are needed for resource-limited sensors.

Third, the sensor devices have space to improve. We attempt to adopt alternative sensing technologies to improve the accuracy and reliability of occupancy detection systems. For example, thermal imaging or infrared sensors could be explored to detect occupancy patterns, as these technologies are not affected by environmental factors such as noise or light. Additionally, wearable or embedded sensors could be explored to provide more accurate and comprehensive data on occupant behavior, which could be used to refine occupancy detection systems further. A more accurate and reliable occupancy detection system could be developed by combining multiple sensing technologies, such as cameras, motion sensors, acoustic sensors, and HVAC sensors. Additionally, a network of sensors and mobile data could be explored and investigated to enhance the system’s accuracy and dependability.

Fourth, occupancy detection algorithms have space to improve. The methods for occupancy detection are still limited and not comprehensive. As one of the planned future works, we will attempt to explore more comprehensive methods for occupancy detection and study their tradeoff. Particularly, we will consider methods considering some factors, such as source-limited sensors, privacy concerns, cost (such as sensor cost, deployment cost, installation cost, maintenance cost, time-consuming cost), environmental conditions (such as lighting and temperature), etc.

Finally, as one of the future works, developing effective Deep Learning (DL)-based occupancy detection solutions necessitates extensive datasets for training and testing DL models. However, such datasets in the literature are currently limited and not widely accessible. To tackle this issue, generating comprehensive datasets that align with DL models’ demands regarding volume and annotation is crucial. This will enable training supervised DL algorithms and simplify comparing their outcomes. Furthermore, exploring collaborative initiatives for sharing resources and establishing standardized datasets could benefit the research community, fostering progress in this field.

## 8. Conclusions

This paper provides a thorough and insightful survey of occupancy detection in smart buildings, encompassing various crucial aspects. An extensive discussion on the types and technologies of sensors used for occupancy detection was presented, highlighting their strengths and weaknesses. This comparison of occupancy detection sensors offered valuable insights into selecting the most suitable sensor technology for specific applications and environments within smart buildings.

Furthermore, the paper delves into integrating these sensors within the broader context of smart buildings. The discussion on IoT system architecture emphasizes sensors’ interconnectedness and their role in creating intelligent and responsive building environments. This insight into the system architecture paves the way for a deeper understanding of how sensors operate in conjunction with other components to optimize building functionality.

Exploring occupancy detection algorithms spans traditional methods and machine learning approaches. Comparing these two paradigms provides valuable insights into their respective strengths and weaknesses. This section equips readers with a nuanced understanding of the algorithmic strategies available, enabling them to make informed decisions based on the specific requirements of their smart building projects.

Additionally, we addressed the challenges inherent in deploying occupancy detection systems, ranging from privacy concerns to the adaptability of algorithms in dynamic environments. These challenges underscore the importance of continued research and innovation in this field. Finally, we outlined several promising areas for future work, including the standardization of benchmarks, the development of privacy-preserving algorithms, the exploration of alternative sensor technologies, and the creation of comprehensive datasets for DL models. These research directions hold the potential to address existing challenges and further enhance the accuracy, efficiency, and cost-effectiveness of occupancy detection in smart buildings.

## Figures and Tables

**Table 1:** Algorithm comparison presented in the survey research papers and our work.

Ref.	Year	Analysis Algorithms Used										
		BOD	WOD	MOD	FLOD	SVM	KNN	RF	DL: FNN	DL: CNN	DL: RNN	DL: LSTM
[17]	2022									✓	✓	✓
[18]	2022					✓	✓			✓		
[19]	2022					✓	✓			✓	✓	
[20]	2020									✓	✓	
[21]	2020	✓				✓	✓					
[22]	2022	✓				✓	✓		✓			
[23]	2019					✓		✓		✓	✓	✓
[24]	2022					✓	✓					
[25]	2015				✓							
[26]	2018	✓				✓	✓	✓				
[27]	2021					✓	✓					
OURS	2024	✓	✓	✓	✓	✓	✓	✓	✓	✓	✓	✓

**Table 2:** Table comparing the four occupancy detection sensors [16].

Sensor Type	Major Analytical Methods	Intrusiveness Level	Sensor Fusion	Accuracy	Occupancy Resolution	Performance Measure
Motion sensors	PIR, ultrasonic, microwave, or a combination	Low because they blend into the environment and do not directly interact with occupants	Can use sensor fusion with other sensors	Can have high accuracy but may be affected by environmental factors	Can distinguish between one or multiple people depending on sensor type	Faster response time, limited detection range (up to 30 feet), energy efficient and consume very little power.
Camera-based sensors	Image processing algorithms that detect human shapes and movements	High due to their visual recording capabilities	Can combine data from multiple cameras	Can have high accuracy but may be affected by lighting and occlusions	Can distinguish between one or multiple people depending on camera resolution	Slower response time, wide detection range, and consume more power due to continuous image capture and processing.
Acoustic sensors	Measure sound waves and patterns to detect human presence	Low to Moderate as they emit sound waves, which occupants might notice. However, they are less intrusive than cameras.	Can use sensor fusion with other sensors	Can have lower accuracy than other sensors but are less affected by environmental factors	Can detect general occupancy but cannot distinguish between one or multiple people	Fast response time, covers larger area for detecting sound range and consume low power.
HVAC sensors	Measure changes in temperature and airflow caused by human presence	Low because they blend into the environment and do not directly interact with occupants	Usually not used with sensor fusion	Can have lower accuracy than other sensors, and may be affected by other factors such as HVAC system settings	Can detect general occupancy but cannot distinguish between one or multiple people	Near-instantaneous response time, detection range depend on the specific parameter measured and it is energy efficient.

**Table 3:** Overview of the usage of sensors in smart buildings [6].

Sensor	Building Type	Centralized/Decentralized	Application System	Energy Saved	Cost
Motion sensors	Commercial/residential	Decentralized	Lightning control	Up to 30%	Low
Camera-based sensors	Commercial	Centralized	Security and surveillance	Up to 20%	High
Acoustic sensors	Commercial/residential	Decentralized	Occupancy detection	Up to 20%	Moderate
HVAC sensors	Commercial	Centralized	Temperature and humidity control	Up to 30%	Moderate

**Table 4:** Advantages, disadvantages, and applications of BOD algorithm in smart buildings.

Advantages	Disadvantages	Applications
Flexibility: It can be adapted to different types of spaces and sensor configurations.	Data requirements: It needs a large amount of data to accurately estimate occupancy state, which may be difficult to obtain in older buildings.	Building energy management: Optimizing heating, cooling, and lighting systems based on occupancy patterns for energy savings and comfort.
Accuracy: It provides accurate occupancy state estimations by considering various factors.	Complexity: It is complex and requires advanced data processing techniques and machine learning algorithms.	Indoor air quality monitoring: Optimizing ventilation systems based on occupancy patterns to improve indoor air quality in schools and offices.
Energy efficiency: It helps optimize energy usage by accurately detecting occupancy states.	Sensitivity to sensor errors: It is sensitive to sensor errors, which can affect occupancy state estimations.	Security monitoring: Detecting unusual occupancy patterns to improve building security and response times to threats.
Cost-effectiveness: It is a cost-effective solution that does not require expensive hardware or complex installations.	Privacy concerns: It raises privacy concerns due to sensor data capturing personal information.	Retail analytics: Analyzing customer traffic patterns in retail stores to optimize layouts and improve customer experience.

**Table 5:** Advantages, disadvantages, and applications of WOD algorithm in smart building.

Advantages	Disadvantages	Applications
Low cost: It is a cost-effective solution using inexpensive motion sensors.	Limited sensing range: It may require multiple sensors for accurate detection in larger spaces.	Smart home automation: Automating home devices based on occupancy and movement for enhanced comfort.
Ease of installation: It is easy to install, requiring minimal hardware and modifications.	Sensitivity to environmental factors: Environmental conditions can impact accuracy.	Health monitoring: Monitoring the walking patterns of the elderly or disabled to detect health abnormalities.
High accuracy: It provides accurate occupancy state estimations based on movement and direction.	False positives: It may generate false positives due to non-human movement detection.	Retail analytics: Tracking customer movements in retail stores to gather behavior data and optimize store layout.
Real-time monitoring: It enables real-time occupancy state monitoring for energy optimization and comfort.	Privacy concerns: Data from motion sensors may raise privacy concerns regarding personal information.	Security monitoring: Detecting and tracking intruders’ movements in a building to enhance security.

**Table 6:** Advantages, disadvantages, and applications of MOD algorithm in smart buildings.

Advantages	Disadvantages	Applications
High accuracy: The MOD algorithm provides more accurate occupancy state estimations using multiple sensors.	High cost: The MOD algorithm requires expensive installation and maintenance of multiple sensors.	Smart building automation: Automating building systems based on occupancy patterns for energy savings and occupant comfort.
Robustness: The MOD algorithm is less sensitive to environmental factors, enhancing accuracy compared to single-sensor algorithms.	Complexity: The MOD algorithm is more complex, making installation and configuration more challenging.	Healthcare monitoring: Monitoring patient movements in hospitals and nursing homes for timely assistance.
Flexibility: The MOD algorithm can be customized to suit different building types and occupancy patterns.	Data management: Effective data management is required due to the substantial amount of data produced by multiple sensors.	Industrial automation: Optimizing production by tracking worker and material movements in manufacturing plants.
Real-time monitoring: The MOD algorithm provides real-time occupancy state monitoring for energy optimization and occupant comfort.	Maintenance: Regular maintenance is needed to ensure sensors provide accurate estimations.	Home security: Detecting and tracking intruders’ movements in homes for enhanced security.

**Table 7:** Advantages, disadvantages, and applications of FLOD algorithm in smart buildings.

Advantages	Disadvantages	Applications
Handling imprecise data: Fuzzy Logic-based Occupancy Detection can effectively handle imprecise and uncertain data, which is common in real-world environments. This capability allows it to make reasonable decisions even when exact information is unavailable or noisy.	Lower accuracy compared to advanced techniques: While fuzzy logic is effective in dealing with uncertainty, it may not achieve the same level of accuracy as some advanced occupancy detection methods, such as machine learning algorithms or Deep Learning-based models.	Energy management in smart buildings: Occupancy detection algorithms based on fuzzy logic can automate building systems such as security, lighting, and HVAC. The algorithm can detect occupancy patterns and adjust building systems accordingly, resulting in energy savings and increased occupant comfort.
Flexibility: The approach offers flexibility in defining input variables and linguistic rules. This adaptability allows the system to accommodate diverse and complex scenarios, making it suitable for various applications.	Complex rule design: Designing fuzzy rules can be time-consuming, especially in more complex applications with numerous input variables and fuzzy sets. Expert knowledge is often required to create effective rules. More challenging.	Healthcare monitoring: Fuzzy logic-based occupancy detection algorithms can be used to monitor patient movements in hospitals and nursing homes. If the algorithm detects that a patient has fallen or is in distress, it will notify healthcare personnel and request immediate assistance.
Robustness to noise: Fuzzy Logic-based Occupancy Detection is robust to noisy sensor readings and fluctuations, enabling it to provide more stable occupancy predictions in dynamic environments.	Interpretability challenges: The interpretability of the fuzzy rules can be challenging, which may hinder understanding the decision-making process in the system.	Vehicle occupancy detection algorithms based on fuzzy logic can be used to detect the presence of passengers in vehicles. This is useful for collecting tolls and monitoring carpool lanes.
Human-like decision making: The method closely mimics human reasoning, making it suitable for applications where human-like decision-making is desired, especially in ambiguous situations.	Performance in noisy environments: Although fuzzy logic is robust to some noise, in extremely noisy environments, the system’s performance may degrade, affecting its reliability.	Industrial automation: Fuzzy logic-based occupancy detection algorithms can be used in manufacturing plants to optimize production by detecting and tracking worker and material movements. This can help to increase efficiency and decrease waste.
Real-time responsiveness: Fuzzy Logic-based Occupancy Detection can make real-time predictions, making it applicable to time-sensitive systems that require immediate occupancy status updates.	Tuning and maintenance: Proper tuning of membership functions and rule sets is essential for optimal performance. Maintenance and updates may also be required to adapt to changing environmental conditions.	Home automation: Occupancy detection algorithms based on fuzzy logic can automate home systems such as lighting, heating, and security. The algorithm can detect occupancy patterns and adjust home systems, accordingly, resulting in energy savings and increased occupant comfort.

**Table 8:** Advantages, disadvantages, and applications of SVM algorithm in smart building.

Advantages	Disadvantages	Applications
It can handle the complexity of occupancy detection in environments with multiple influencing factors (e.g., temperature, humidity, light) that affect occupancy. Good at handling complex decision boundaries.	It can be computationally demanding, leading to longer training times and potential hardware requirements, especially with large datasets.	It can be used in smart building systems to detect occupancy in various rooms and spaces. It can determine whether a room is occupied by analyzing temperature, humidity, and motion sensor data.
It creates clear boundaries between occupied and unoccupied spaces, even in cases of overlapping data, making it effective for binary classification.	Its performance relies on choosing the right hyperparameters, like the regularization parameter (C) and kernel function. This requires careful tuning.	This information can be used to optimize heating, cooling, lighting, and ventilation systems, leading to energy savings.
It performs well even with noisy data, making it suitable for real-world occupancy detection where sensor data may have inaccuracies.	It is naturally suited for binary classification, so adapting it for occupancy detection scenarios with more than two states (e.g., unoccupied, occupied, partially occupied) can be complex.	It can trigger alarms or notifications when unexpected occupancy patterns are detected, which can be valuable in security, safety, and emergency response systems.

**Table 9:** Advantages, disadvantages, and applications of KNN algorithm in smart buildings.

Advantage	Disadvantage	Applications
As a non-parametric approach, it makes no assumptions about data distribution, making it suitable for complex and nonlinear occupancy detection scenarios. Its versatility allows it to capture local patterns effectively, providing valuable insights into specific spatial or temporal occupancy patterns in buildings or spaces.	It does not handle irrelevant features well. In occupancy detection, it is essential to carefully choose and preprocess features to avoid noise in the data.	It can be used for presence detection in smart homes, triggering automated lighting, heating, and security systems based on whether rooms are occupied or unoccupied.
It is Intuitive and easy to implement.	It is sensitive to the choice of K and distance metric; Poor choices can lead to suboptimal results.	It can monitor crowd density and occupancy levels for security and resource management.
It can adapt to changing occupancy patterns as it continuously learns from incoming data. This adaptability is useful for occupancy detection in dynamic environments.	It may be computationally expensive With huge datasets or high-dimensional feature spaces. It can take some time to determine the distances to all data points.	It can help optimize lighting systems in buildings by adjusting light intensity based on the number of people in a room, contributing to energy savings.

**Table 10:** Advantages, disadvantages, and applications of RF algorithm in smart buildings.

Advantage	Disadvantage	Applications
It frequently offers good accuracy in tasks involving occupancy detection. It is renowned for its capacity to reduce overfitting and model complex relationships in data. It can handle categorical characteristics and high-dimensional data.	While Random Forest offers high accuracy, its ensemble nature makes it less interpretable than individual decision trees. Understanding how the model makes decisions can be challenging.	It can optimize energy usage in buildings by predicting occupancy and controlling heating, cooling, and lighting systems accordingly, leading to energy savings.
It mitigates overfitting by aggregating multiple decision trees. This makes it suitable for noisy datasets common in occupancy detection scenarios.	Training a Random Forest with many trees can be computationally intensive and time-consuming, especially with extensive datasets.	It can assist security systems by detecting unauthorized access or intrusions based on occupancy patterns, sensor data, and motion detection.
It can handle datasets with numerous features, accommodating the multiple sensors often used in occupancy detection systems.	Using more trees in a Random Forest can consume significant memory, which can be a limitation on resource-constrained systems.	It can be applied to adjust lighting levels in response to occupancy changes dynamically, ensuring efficient use of electricity in commercial and residential spaces.

**Table 11:** Advantages, disadvantages, and applications of FNN in smart buildings.

Advantages	Disadvantages	Applications
FNNs are computationally efficient, making them suitable for real-time applications in smart building systems.	FNNs do not inherently handle sequential data or time-dependent patterns, which is essential in occupancy detection tasks.	FNNs can be used for binary occupancy detection tasks, where the goal is to guess whether a space is occupied or not
FNN can capture complicated non-linear relationships between input features, allowing for accurate occupancy predictions.	FNNs do not have memory of past inputs, which is crucial for tasks where temporal dependencies matter.	They can predict when maintenance is needed based on occupancy patterns, helping to prevent system failures.
They can be easily scaled to handle large datasets, making them adaptable to different building environments.	Without proper regularization techniques, FNNs can overfit the training data, leading to poor generalization of new data.	They can assist in optimizing energy usage in smart buildings by predicting occupancy patterns and adjusting HVAC systems accordingly.
FNNs can generalize well to new, unseen data, provided they are properly trained and not overfitted.	Extracting relevant features from raw data might require domain expertise, and the effectiveness of features depends on the engineer’s knowledge.	They can be easily scaled to handle large datasets, making them adaptable to different building environments.

**Table 12:** Advantages, disadvantages, and applications of CNN in smart buildings.

Advantages	Disadvantages	Applications
CNNs are great at capturing spatial hierarchies in data, which makes them perfect for processing grid-like data, such as pictures and occupancy grid maps.	CNNs are specifically designed for grid-like data, which may limit their applicability in tasks that involve sequential or non-grid data.	CNNs can process images from cameras to detect occupancy in smart buildings, making them suitable for security and energy optimization applications.
CNNs can recognize patterns regardless of their position in the input, which is useful in occupancy detection jobs where the spatial arrangement of sensors may vary.	CNN architectures can be complex, requiring careful design and tuning of hyperparameters.	They can process occupancy grid maps, common representations of spaces in smart building environments, to predict occupancy patterns.
In smart buildings, CNNs can process images from cameras and other sensors, extracting valuable information for occupancy detection.	The internal workings of CNNs may be less interpretable than simpler models like FNNs.	CNNs can be used for facial recognition systems, which can be integrated into access control systems for occupancy verification.

**Table 13:** Advantages, disadvantages, and applications of RNN in smart buildings.

Advantages	Disadvantages	Applications
RNNs are specifically designed for processing sequential data, making them ideal for tasks involving time series information, which is common in occupancy detection.	Due to the vanishing or inflating gradient problem, training RNNs can be difficult, especially for deep networks or lengthy sequences.	RNNs are well-suited for predicting future occupancy based on historical data, making them valuable for energy optimization in smart buildings.
They can capture temporal dependencies and model how occupancy patterns evolve.	RNNs can be computationally intensive, which may lead to longer training times compared to simpler models.	They can classify activities based on sensor data sequences, helping to infer occupancy patterns in different building areas.
RNNs can handle sequences of varying lengths, which is crucial in occupancy detection where the duration of data collection may differ.	Standard RNNs may struggle to capture long-range dependencies, which may be crucial in some occupancy detection scenarios.	They can model occupants’ behavior over time, enabling the prediction of occupancy patterns and optimizing building systems.

**Table 14:** Advantages, disadvantages, and applications of LSTMs in smart buildings.

Advantages	Disadvantages	Applications
LSTMs are designed to capture long-range dependencies in sequential data, which is crucial for modeling occupancy patterns that evolve over extended periods.	LSTMs can be computationally intensive, which may lead to longer training times compared to simpler models.	LSTMs are well-suited for predicting future occupancy based on historical data, making them valuable for energy optimization in smart buildings
LSTMs can retain and utilize information from earlier time steps, making them effective for tasks where historical context is important.	Properly configuring an LSTM network with appropriate hyperparameters can be challenging. It may require some expertise	They can identify unusual patterns or events in occupancy data, which is crucial for security and safety applications.
They are highly effective for processing time series data, which is common in occupancy detection tasks in smart buildings.	Without proper regularization techniques, LSTMs can overfit to the training data, leading to poor generalization on new data	LSTMs can model the behavior of occupants over time, enabling the prediction of occupancy patterns and optimizing building systems

**Table 15:** Compares BOD, FLOD, MOD, and WOD occupancy detection algorithms.

Algorithms	PIR	Environmental Sensors	Accuracy	Smart Meters	Sensor Fusion
BOD	High	Low	High	No	No
FLOD	Low	Medium	High	No	Yes
MOD	Low	High	Medium	Yes	Yes
WOD	Medium	Low	Low	Yes	No

**Table 16:** Compares SVM, KNN, RF, and DL occupancy detection algorithms.

Algorithm	Accuracy	Training Time	Memory Requirement	Scalability	Robustness
SVM	High	Low	High	Low	High
KNN	Medium	Low	High	High	Low
Random Forest	High	Medium	High	High	High
Deep Learning	High	High	High	High	High

**Table 17:** Comparison of the performances of applying SVM, KNN, and RF to the occupancy data.

Metric	SVM	KNN	Random Forest
Accuracy	0.85	0.88	0.90
Precision	0.87	0.86	0.91
Recall	0.82	0.89	0.94
F1-Score	0.84	0.87	0.92
ROC-AUC	0.91	0.92	0.96

**Table 18:** Comparison of the performances of applying FNN, CNN, RNN, and LSTM to the occupancy data.

Metric	FNN	CNN	RNN	LSTM
Accuracy	0.89	0.91	0.88	0.93
Precision	0.88	0.90	0.87	0.92
Recall	0.90	0.92	0.89	0.94
F1-Score	0.89	0.91	0.88	0.93
ROC-AUC	0.94	0.95	0.93	0.96

## Data Availability

Data are contained within the article.

## References

[1] Zhou K., Yang S. (2018). 5.11 Smart Energy Management. Comprehensive Energy Systems.

[2] Smart Buildings Market Worth 121.6 Billion Dollars by 2026-Report by Marketsandmarkets. https://www.marketsandmarkets.com/PressReleases/smartbuilding.asp.

[3] The Edge, Amsterdam: Showcasing an Exemplary IoT Building. https://www.cdbb.cam.ac.uk/system/files/documents/TheEdge_Paper_LOW1.pdf.

[4] Asim N., Badiei M., Mohammad M., Razali H., Rajabi A., Chin Haw L., Jameelah Ghazali M. (2022). Sustainability of Heating, Ventilation and Air-conditioning (HVAC) Systems in Buildings—An Overview. Int. J. Environ. Res. Public Health.

[5] Auffenberg F., Snow S., Stein S., Rogers A. (2017). A Comfort-based Approach to Smart Heating and Air Conditioning. ACM Trans. Intell. Syst. Technol..

[6] Akkaya K., Guvenc I., Aygun R., Pala N., Kadri A. Iot-based Occupancy Monitoring Techniques for Energy-efficient Smart Buildings. Proceedings of the 2015 IEEE Wireless Communications and Networking Conference Workshops.

[7] Chasta R., Singh R., Gehlot A., Mishra R., Choudhury S. (2016). A Smart Building Automation System. Int. J. Smart Home.

[8] Murali M., Ramsami A., Varanasi S. (2019). Smart Building Automation Using Internet of Things. IRJET J..

[9] Statista Smart and Connected Home Market Revenue Worldwide in 2019 and Forecast for 2025. https://www.statista.com/statistics/805213/building-energy-management-system-market-worldwide/.

[10] Taunk K., De S., Verma S., Swetapadma A. A Brief Review of Nearest Neighbor Algorithm for Learning and Classification. Proceedings of the 2019 International Conference on Intelligent Computing and Control Systems (ICCS).

[11] Hernández J.L., Sanz R., Corredera Á., Palomar R., Lacave I. (2018). A Fuzzy-based Building Energy Management System for Energy Efficiency. Buildings.

[12] Whitmore A., Agarwal A., Da Xu L. (2014). The Internet of Things—A Survey of Topics and Trends. Inf. Syst. Front..

[13] Ding Y., Chen W., Wei S., Yang F. (2021). An Occupancy Prediction Model for Campus Buildings Based on the Diversity of Occupancy Patterns. Sustain. Cities Soc..

[14] Jeon Y., Cho C., Seo J., Kwon K., Park H., Oh S., Chung I.-J. (2018). Iot-based Occupancy Detection System in Indoor Residential Environments. Build. Environ..

[15] Luo L., Xiao Y., Liang W. Encoding Space to Count Multi-targets with Multiplexed Binary Infrared Sensors. Proceedings of the 2019 15th International Conference on Mobile Ad-Hoc and Sensor Networks (MSN).

[16] Zhang H., Davigny A., Colas F., Poste Y., Robyns B. (2012). Fuzzy Logic Based Energy Management Strategy for Commercial Buildings Integrating Photovoltaic and Storage Systems. Energy Build..

[17] What Is Statistical Modeling? When and Where to Use It. https://www.g2.com/articles/statistical-modeling,.

[18] Zhao L., Li Y., Liang R., Wang P. (2022). A State of Art Review on Methodologies of Occupancy Estimating in Buildings from 2011 to 2021. Electronics.

[19] Tien P.W., Wei S., Darkwa J., Wood C., Calautit J.K. (2022). Machine Learning and Deep Learning Methods for Enhancing Building Energy Efficiency and Indoor Environmental Quality—A Review. Energy AI.

[20] Akinosho T.D., Oyedele L.O., Bilal M., Ajayi A.O., Delgado M.D., Akinade O.O., Ahmed A.A. (2020). Deep learning in the construction industry: A review of present status and future innovations. J. Build. Eng..

[21] Rueda L., Agbossou K., Cardenas A., Henao N., Kelouwani S. (2020). A comprehensive review of approaches to building occupancy detection. Build. Environ..

[22] Zhang W., Wu Y., Calautit J.K. (2022). A review on occupancy prediction through machine learning for enhancing energy efficiency, air quality and thermal comfort in the built environment. Renew. Sustain. Energy Rev..

[23] Saha H., Florita A.R., Henze G.P., Sarkar S. (2019). Occupancy sensing in buildings: A review of data analytics approaches. Energy Build..

[24] Turgut Z., Akgün G. Occupancy detection for energy efficiency of smart buildings using machine learning. Proceedings of the 19th International Conference on Sustainable Energy Technologies.

[25] Rafsanjani H.N., Ahn C.R., Alahmad M. (2015). A Review of Approaches for Sensing, Understanding, and Improving Occupancy-Related Energy-Use Behaviors in Commercial Buildings. Energies.

[26] Chen Z., Jiang C., Xie L. (2018). Building occupancy estimation and detection: A review. Energy Build..

[27] Kanthila C., Boodi A., Beddiar K., Amirat Y., Benbouzid M. (2021). Building Occupancy Behavior and Prediction Methods: A Critical Review and Challenging Locks. IEEE Access.

[28] Peng M., Xiao Y., Li N., Liang X. (2014). Monitoring Space Segmentation in Deploying Sensor Arrays. IEEE Sens. J..

[29] Shih O., Lazik P., Rowe A. Aures: A Wide-band Ultrasonic Occupancy Sensing Platform. Proceedings of the 3rd ACM International Conference on Systems for Energy-Efficient Built Environments.

[30] Rahman H., Han H. (2018). Bayesian Estimation of Occupancy Distribution in a Multi-room Office Building Based on CO_2_ Concentrations. Build. Simul..

[31] Chițu C., Stamatescu G., Stamatescu I., Sgârciu V. Wireless System for Occupancy Modelling and Prediction in Smart Buildings. Proceedings of the 25th Mediterranean Conference on Control and Automation.

[32] Mahanta M., Taparugssanagorn A., Pati B.M. (2022). Assessment of spectrum sensing using support vector machine combined with principal component analysis. Int. J. Sens. Netw..

[33] Surasakhon W., Taparugssanagorn A., Lerspalungsanti S., Thipprachak K. (2022). Early detection of a fall using Wi-Fi and deep learning. Int. J. Sens. Netw..

[34] Filippoupolitis A., Oliff W., Loukas G. Bluetooth Low Energy Based Occupancy Detection for Emergency Management. Proceedings of the 2016 15th International Conference on Ubiquitous Computing and Communications and 2016 International Symposium on Cyberspace and Security (IUCC-CSS).

[35] Tekler Z.D., Low R., Gunay B., Andersen R.K., Blessing L. (2020). A scalable Bluetooth Low Energy approach to identify occupancy patterns and profiles in office spaces. Build. Environ..

[36] Luo L., Xiao Y., Liang W. (2021). The Maximum Number of Cells with Modulated Binary Sensors. IEEE Sens. J..

[37] Elkhoukhi H., NaitMalek Y., Berouine A., Bakhouya M., Elouadghiri D., Essaaidi M. (2018). Towards a Real-time Occupancy Detection Approach for Smart Buildings. Procedia Comput. Sci..

[38] Luo L., Xiao Y., Liang W., Zheng M. (2020). A Cell Reconstruction Tool to Deploy Binary Pyroelectric Sensor Arrays. IEEE Sens. J..

[39] Demrozi F., Turetta C., Chiarani F., Kindt P.H., Pravadelli G. (2021). Estimating indoor occupancy through low-cost BLE devices. IEEE Sens. J..

[40] Mateos Sánchez M., Berjón Gallinas R., Beato Gutiérrez M.E., Fermoso García A.M. (2020). A Tool to Calculate the Level of Occupancy in Indoor and Outdoor Spaces Using BLE and Open Data to Be Published in Real-Time. Sensors.

[41] Leitch S.G., Ahmed Q.Z., Abbas W.B., Hafeez M., Laziridis P.I., Sureephong P., Alade T. (2023). On Indoor Localization Using WiFi, BLE, UWB, and IMU Technologies. Sensors.

[42] Heidary R., Prasad Rao J., Pinon Fischer O.J., Fathi M., Zio E., Pardalos P.M. (2023). Smart Buildings in the IoT Era—Necessity, Challenges, and Opportunities. Handbook of Smart Energy Systems.

[43] Thieme W. Smart Building Communications: Three Building Blocks Explained. https://www.forbes.com/sites/forbestechcouncil/2021/01/06/smart-building-communications-three-building-blocks-explained/.

[44] Xiao Y. (2005). IEEE 802.11 Performance Enhancement via Concatenation and Piggyback Mechanisms. IEEE Trans. Wirel. Commun..

[45] Spathi K.P., Beletsioti G.A., Kantelis K.F., Valkanis A., Nicopolitidis P., Papadimitriou G.I. (2023). Increasing device energy efficiency in LoRaWAN networks via a learning-automata-based approach. Int. J. Sens. Netw..

[46] King J., Perry C. Smart Buildings: Using Smart Technology to Save Energy in Existing Buildings. https://www.aceee.org/sites/default/files/publications/researchreports/a1701.pdf.

[47] Wang C., Jiang J., Roth T., Nguyen C., Liu Y., Lee H. (2021). Integrated Sensor Data Processing for Occupancy Detection in Residential Buildings. Energy Build..

[48] Amayri M., Ploix S., Kazmi H., Ngo Q.-D., Safadi E. (2019). Estimating Occupancy from Measurements and Knowledge Using the Bayesian Network for Energy Management. J. Sens..

[49] Bapin Y., Zarikas V. (2019). Smart Building’s Elevator with Intelligent Control Algorithm Based on Bayesian Networks. Int. J. Adv. Comput. Sci. Appl..

[50] Youssef Y. (2022). Bayes Theorem and Real-Life Applications. Ph.D. Thesis.

[51] Kontou E., Stylianides N. (2020). Bayes Theorem and Its Recent Applications. Math. Res. J..

[52] Singh A., Kansal V., Gaur M., Pandey M.S. Predicting Smart Building Occupancy Using Machine Learning. Proceedings of the Third Doctoral Symposium on Computational Intelligence.

[53] Drira S., Pai S.G.S., Reuland Y., Olsen N.F.H., Smith I.F.C. (2021). Using Footstep-induced Vibrations for Occupant Detection and Recognition in Buildings. Adv. Eng. Informat..

[54] Chen L., Chen F., Guan X. (2009). A Video-based Indoor Occupant Detection and Localization Algorithm for Smart Buildings. Proceedings of the Emerging Intelligent Computing Technology and Applications: 5th International Conference on Intelligent Computing.

[55] Shih H.-C. (2014). A Robust Occupancy Detection and Tracking Algorithm for the Automatic Monitoring and Commissioning of a Building. Energy Build..

[56] Sarkar S., Ghosh A., Chatterjee S. (2022). Multi-sensor Data Fusion for Occupancy Detection Using Dempster—Shafer Theory. Computational Intelligence in Data Mining: Proceedings of ICCIDM.

[57] Rajabi H., Hu Z., Ding X., Pan S., Du W., Cerpa A. MODES: Multi-sensor Occupancy Data-driven Estimation System for Smart Buildings. Proceedings of the Thirteenth ACM International Conference on Future Energy Systems.

[58] Xu S., Hou Y., Deng X., Chen P., Ouyang K., Zhang Y. (2021). A Novel Divergence Measure in Dempster–shafer Evidence Theory Based on Pignistic Probability Transform and Its Application in Multi-sensor Data Fusion. Int. J. Distrib. Sens. Netw..

[59] Su X., Dong Z. A Modified Combination Rule to Conflict Evidence for Dempster-Shafer Theory. Proceedings of the 2015 International Conference on Computational Intelligence and Communication Networks (CICN).

[60] Ding Y., Han S., Tian Z., Yao J., Chen W., Zhang Q. (2022). Review on Occupancy Detection and Prediction in Building Simulation. Build. Simul..

[61] Baniyounes A. (2019). Energy Performance of Fuzzy Logic Controllers in Smart Buildings. Int. J. Energy Econ. Policy.

[62] Escobar L.M., Aguilar J., Garcés-Jiménez A., De Mesa J.A.G., Gomez-Pulido J.M. (2020). Advanced Fuzzy-logic-based Con-text-driven Control for HVAC Management Systems in Buildings. IEEE Access.

[63] Boultache T., Achour B., Laghrouche M. (2022). Human activity detection from inertial data using RNN and LSTM network. Int. J. Sens. Netw..

[64] Toutiaee M. (2021). Occupancy Detection in Room Using Sensor Data. arXiv.

[65] Sayed A.N., Himeur Y., Bensaali F. (2022). Deep and Transfer Learning for Building Occupancy Detection: A Review and Comparative Analysis. Eng. Appl. Artif. Intell..

[66] Kim M.-L., Park K.-J., Son S.-Y. (2023). Occupancy-based Energy Consumption Estimation Improvement through Deep Learning. Sensors.

[67] Mitra D., Malekpour Koupaei D., Cetin K. (2022). Occupancy Data Sensing, Collection, and Modeling for Residential Buildings. Renewable Energy for Buildings: Technology, Control, and Operational Techniques.

[68] Chen Z., Wang Y., Liu H. (2018). Unobtrusive Sensor-based Occupancy Facing Direction Detection and Tracking Using Advanced Machine Learning Algorithms. IEEE Sens. J..

[69] Murray D.G., Simsa J., Klimovic A., Indyk I.T. (2021). Data: A Machine Learning Data Processing Framework. arXiv.

[70] Cervantes J., Garcia-Lamont F., Rodríguez-Mazahua L., Lopez A. (2020). A Comprehensive Survey on Support Vector Machine Classification: Applications, Challenges and Trends. Neurocomputing.

[71] Boateng E., Otoo J., Abaye D. (2020). Basic Tenets of Classification Algorithms K-Nearest-Neighbor, Support Vector Machine, Random Forest and Neural Network: A Review. J. Data Anal. Inf. Process..

[72] Al-Habashna A., Wainer G., Aloqaily M. (2022). Machine learning-based indoor localization and occupancy estimation using 5G ultra-dense networks. Simul. Model. Pract. Theory.

[73] Wu L., Wang Y. (2021). Stationary and Moving Occupancy Detection Using the SLEEPIR Sensor Module and Machine Learning. IEEE Sens. J..

[74] Parzinger M., Hanfstaengl L., Sigg F., Spindler U., Wellisch U., Wirnsberger M. (2022). Comparison of Different Training Data Sets from Simulation and Experimental Measurement with Artificial Users for Occupancy Detection—Using Machine Learning Methods Random Forest and LASSO. Build. Environ..

[75] Li X., Chen W., Zhang Q., Wu L. (2020). Building Auto-encoder Intrusion Detection System Based on Random Forest Feature Selection. Comput. Secur..

[76] Lork C., Li W.-T., Qin Y., Zhou Y., Yuen C., Tushar W., Saha T.K. (2020). An Uncertainty-aware Deep Reinforcement Learning Framework for Residential Air Conditioning Energy Management. Appl. Energy.

[77] Feng C., Mehmani A., Zhang J. (2020). Deep Learning-based Real-time Building Occupancy Detection Using AMI Data. IEEE Trans. Smart Grid.

[78] Becker V., Kleiminger W. (2018). Exploring Zero-training Algorithms for Occupancy Detection Based on Smart Meter Measurements. Comput. Sci. Res. Dev..

[79] Zheng T., Chen Z., Ding S., Luo J. (2021). Enhancing RF Sensing with Deep Learning: A Layered Approach. IEEE Commun. Mag..

[80] Wei Y., Xia L., Pan S., Wu J., Zhang X., Han M., Zhang W., Xie J., Li Q. (2019). Prediction of occupancy level and energy consumption in office building using blind system identification and neural networks. Appl. Energy.

[81] Tekler Z.D., Chong A. (2022). Occupancy Prediction Using Deep Learning Approaches Across Multiple Space Types: A Minimum Sensing Strategy. Build. Environ..

[82] Amato G., Carrara F., Falchi F., Gennaro C., Meghini C., Vairo C. (2017). Deep learning for decentralized parking lot occupancy detection. Expert Syst. Appl..

[83] Zhang Z., Feng F., Huang T. (2022). FNNS: An Effective Feedforward Neural Network Scheme with Random Weights for Processing Large-scale Datasets. Appl. Sci..

[84] Zhang Y., Wu J., Xie H., Hua R., Li Q. (2024). NASA space station rolling bearings anomaly detection based on PARA-LSTM model. Int. J. Sens. Netw..

[85] Zhao H., Qi Z., Wang S., Vafai K., Wang H., Chen H., Tan S.X.-D. Learning-based occupancy behavior detection for smart buildings. Proceedings of the 2016 IEEE International Symposium on Circuits and Systems (ISCAS).

[86] Zhang Y., Zhao L., Fang Y., Mu D., Zhang H. (2023). LSTM-based multi-PIR sensor information fusing for estimating the speed and position of pedestrians on green campus. Int. J. Sens. Netw..

[87] Asiri S., Xiao Y., Alzahrani S., Li S., Li T. (2023). A Survey of Intelligent Detection Designs of HTML URL Phishing Attacks. IEEE Access.

[88] Colace S., Laurita S., Spezzano G., Vinci A. (2022). Room Occupancy Prediction Leveraging LSTM: An Approach for Cognitive and Self-adapting Buildings. IoT Edge Solutions for Cognitive Buildings.

[89] Tao Z., Chen L., Guo X., Li J., Guo J., Liu Y. (2023). Gaussian fitting based human activity recognition using Wi-Fi signals. Int. J. Sens. Netw..

